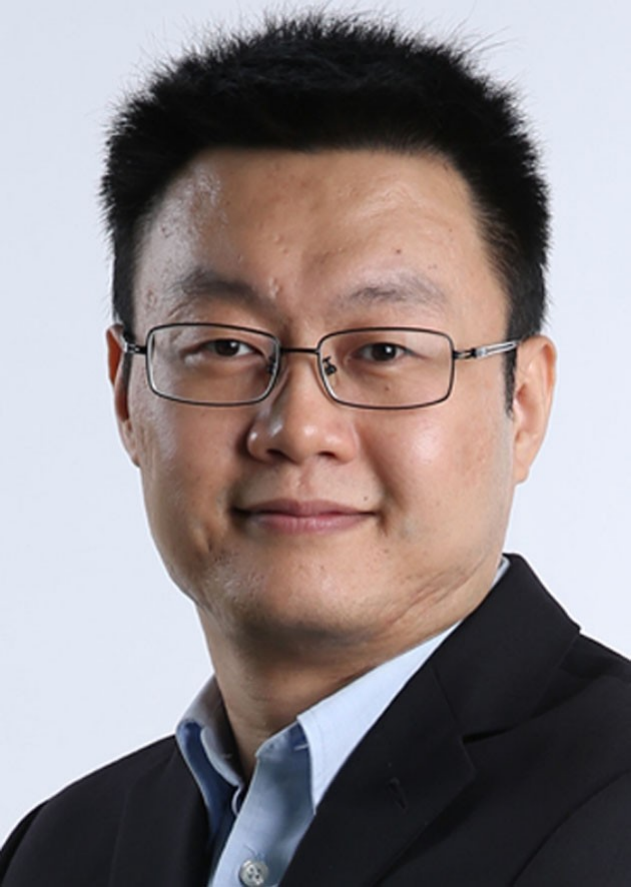# Electrochemistry in Magnetic Fields

**DOI:** 10.1002/anie.202203564

**Published:** 2022-05-25

**Authors:** Songzhu Luo, Kamal Elouarzaki, Zhichuan J. Xu

**Affiliations:** ^1^ School of Materials Science and Engineering Nanyang Technological University 50 Nanyang Avenue Singapore 639798 Singapore; ^2^ Nanyang Environment and Water Research Institute (NEWRI) Interdisciplinary Graduate School 1 Cleantech Loop, CleanTech One Singapore 637141 Singapore; ^3^ Energy Research Institute @ Nanyang Technological University, ERI@N Interdisciplinary Graduate School 50 Nanyang Avenue Singapore 639798 Singapore

**Keywords:** Electrocatalysis, Electrochemistry, Magnetic Field, Magnetic Hyperthermia, Magnetohydrodynamics

## Abstract

Developing new strategies to advance the fundamental understanding of electrochemistry is crucial to mitigating multiple contemporary technological challenges. In this regard, magnetoelectrochemistry offers many strategic advantages in controlling and understanding electrochemical reactions that might be tricky to regulate in conventional electrochemical fields. However, the topic is highly interdisciplinary, combining concepts from electrochemistry, hydrodynamics, and magnetism with experimental outcomes that are sometimes unexpected. In this Review, we survey recent advances in using a magnetic field in different electrochemical applications organized by the effect of the generated forces on fundamental electrochemical principles and focus on how the magnetic field leads to the observed results. Finally, we discuss the challenges that remain to be addressed to establish robust applications capable of meeting present needs.

## Graphical abstract

Conducting electrochemistry in an applied magnetic field generates forces that affect the electrochemical system due to a change in fundamental electrochemical principles. The fundamental mechanisms behind the observed changes in different applications are discussed in this Review. Suggestions on interference exclusion and possible future perspectives of magnetoelectrochemistry are also proposed.

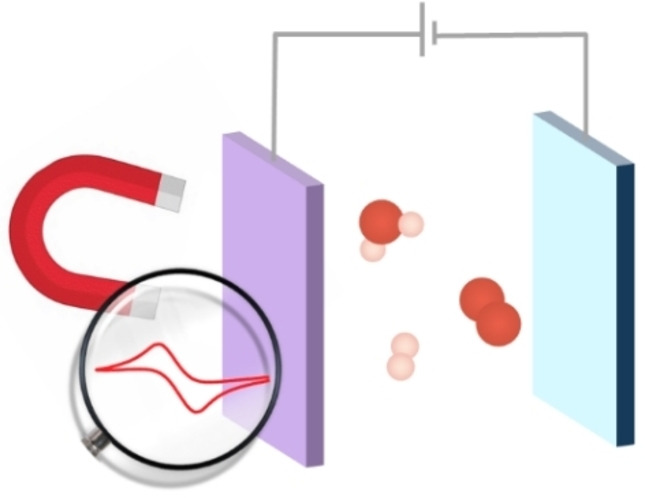

## 1 Introduction

Industry is estimated to account for more than 24 % of the world's annual greenhouse gas (GHG) emissions, with the chemical industry a major contributor to these figures.^[1]^ A key industrial challenge to achieving 2050 climate targets is transitioning from current chemical technologies to more sustainable and carbon‐neutral approaches.^[2]^ Electrifying the chemical industry will have a substantial global impact.^[3]^ Therefore, this sector requires new thinking to replace current carbon‐intensive infrastructures and industrial processes approaching their practical performance limits.^[4]^ Advanced, disruptive, and enabling technologies are required beyond current incremental manufacturing improvements.^[5]^

Electrochemistry is a promising tool for designing various greenhouse gas mitigation technologies that shift chemical industrial operations.[^3b^, ^6^] In particular, the growth of renewable electricity sources both on‐ and off‐grid associated with electrochemical methods provides unique opportunities to move current chemical processes away from traditional high‐carbon and high‐energy reaction pathways.^[7]^ Advances in electrochemistry depend on a better fundamental understanding of complex reactions/phenomena at the electrodes. They require exquisite control of reaction parameters, controlled electrode architectures, and new directions in the design and scaling of electrochemical reactors. New green technologies and processes could emerge considering breakthroughs in fundamental and applied research in electrochemistry. The most promising direction might be to investigate events at the electrodes on atomic‐scale research that could lead to new understandings of catalytic processes and enhanced performance.^[8]^

Among these research directions, magnetoelectrochemistry has recently become an attractive fundamental topic for boosting the overall electrocatalytic performance.^[9]^ An applied magnetic field is a powerful tool to control electrochemical reactions in multiple ways. Unlike high‐temperature and high‐pressure strategies requiring extreme experimental conditions, a commercial magnet is sufficient to boost many chemical reaction features. Interestingly, the influence of an applied magnetic field on the electrochemical process has been investigated for nearly one century.^[10]^ The topic is a cross‐disciplinary approach involving electrochemistry, hydrodynamics, and magnetism phenomena with experimental outcomes that are sometimes unexpected. However, their elucidation can lead to technological breakthroughs opening significant opportunities for developing electrochemical applications. Generally, the magnetic field can influence the complex electrocatalytic system through many effects. For example, the Lorentz force and the Kelvin force can directly act on electric currents and paramagnetic species in the electrolyte, respectively. In addition, the magnetic hyperthermia effect caused by the alternating magnetic field may lead to localized intense heating, facilitating the electrochemical reactions. Other magnetic‐field‐induced effects, such as the Maxwell stress effect and the spin cselectivity effect, may also impact the electrochemical proprieties.

Although the magnetic field has been applied to facilitate electrochemical reactions in some works, significant gaps still exist, especially in the underlying mechanisms that are not well understood or still under debate. From this perspective, this Review provides insights on recently published papers coupling electrochemistry with a magnetic field and discusses how a magnetic field could affect an electrochemical system. Also, we discuss the fundamental mechanism behind any observed changes in different applications. Furthermore, reported works are concerned with a detailed explanation of the complicated mechanism‐related concepts. Finally, we highlight new applications that could be developed considering fundamental and applied results in this field.

Owing to the complexity of this topic, we first introduce magnetically induced forces. The most widely utilized magnetism‐induced Lorentz and Kelvin forces are briefly introduced, and the differences and applicable conditions are discussed. Secondly, we discuss the magnetic hyperthermia effect and its applications when alternating magnetic fields are applied. Next, magnetohydrodynamics (MHD) is thoroughly detailed, using examples in electrodeposition, electrocatalysis, and battery applications. Then, the magnetic‐field‐facilitated spin selectivity effect can be used as a promotion strategy, and the applications in various electrocatalytic reactions are introduced. It should be noted that these magnetic effects are not exclusive and can coexist simultaneously; some suggestions on interference exclusion are given to help researchers obtain concrete conclusions. Lastly, the magnetic‐field‐inspired electrode design strategy and possible exploration areas are proposed.

## 2 Two Dominating Forces in a Magnetic Field: Lorentz Force and Kelvin Force

As mentioned above, the Lorentz and the Kelvin forces are the most fundamental and well‐studied magnetic effects. Coey's group has previously made detailed discussions about these two forces in two short reviews.^[11]^ Here we provide the essential physics background and discuss their difference in an electrochemical system.

When a magnetic field *B* is applied to an electrochemical cell, the field acts on the charged species with the current density *j* to generate the Lorentz force *F*_L_. The relation between the *B*, *j*, and *F*_L_ is given by Equation 1.

(1) $$F_{L}=Bj$$

**Figure 1:**
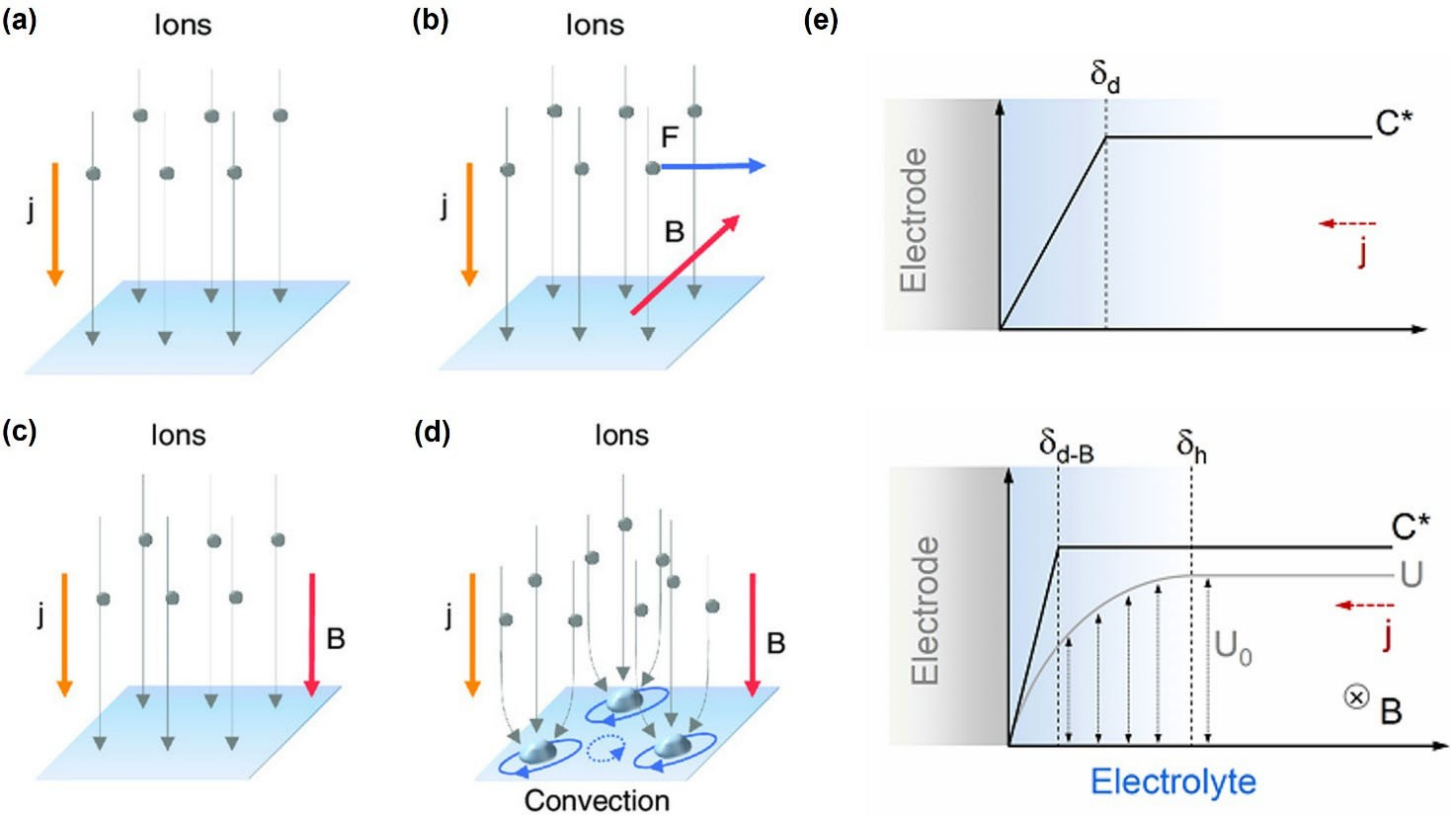
The relation between the magnetic field *B*, current density *j*, and Lorentz force *F*_L_. a) The current distribution in an ideal flat electrode surface; b) *B* is parallel to the electrode while *j* is normal to the ideal electrode; c) *B* and *j* are normal to the electrode; d) The micro rim‐flow around the protrusions and bubbles on the electrode, *B* and *j* are normal to the electrode.^[14]^ Reproduced with permission. Copyright 2020, Wiley‐VCH. e) Schematic of the magnetic‐field‐enhanced mass transport reduction of the thickness of the diffusion layer. In this schematic, C* denotes the concentration of electroactive species as a function of the distance from the electrode surface, *j* is the current flow, δ_d_ is the thickness of the diffusion layer without an applied magnetic field. When a magnetic field is applied, a tangent flow *U* is induced, which leads to the formation of a hydrodynamic boundary layer with a thickness of δ_h_. The velocity changes from *U*_0_ (the value in the bulk stream) to zero within the hydrodynamic boundary layer.^[15]^ Reproduced with permission. Copyright 2022, Dalian Institute of Chemical Physics, the Chinese Academy of Sciences.

It is noteworthy that *B*, *j*, and *F*_L_ are all vectors rather than scalars, representing both magnitude (i.e., module) and directions. The direction of *F*_L_ is perpendicular to the co‐plane of *B* and *j*, determined by the well‐known Fleming's Left‐Hand Rule, as shown in Figure 1b.^[12]^ The module of *F*_L_ is determined by the module of *B* and *j*, and their angle *θ*; when *B* and *j* are orthogonal, the module of *F*_L_ has the highest value. In experimental conditions, the current density is nonuniform at the electrode's edges, which leads to rim‐flow caused by the Lorentz force. Meanwhile, the bulges and entangled bubbles may also lead to similar rim‐flow, as shown in Figure 1d.

The magnetic field is usually uniform and macroscopical when the Lorentz force is discussed and analyzed. Unlike Lorentz force, Kelvin force usually acts on paramagnetic species in the electrolyte rather than the charged species. Kelvin force is generated by a nonuniform magnetic field, which draws the paramagnetic species towards higher magnetic density. The magnitude of Kelvin force is given by Equation 2,

(2) $$F_{K}=\left(\frac{1}{2}\mu_{0}\right)c\chi_{m}\nabla B^{2}$$

where *F*_K_ is the Kelvin force, *μ*_0_ is the magnetic constant (4π×10^−7^ T mA^−1^), *c* is the concentration of magnetic species in mol m^−3^, χ_m_ is the molar susceptibility, and ∇*B* ^2^=2 (*B*∇)*B*, where ∇ is the magnetic field gradient. This equation shows that the Kelvin force is proportional to the magnitude of the magnetic field, the paramagnetic species’ concentration, and the magnetic field gradient. Therefore, the Kelvin force is also called the magnetic field gradient force because the magnetic field gradient generates this force. These two forces originate from the well‐known magnetohydrodynamic (MHD) effects generating convection in the electrolyte macroscopically and microscopically. In 2004, Willner and co‐authors proposed a theoretical hydrodynamic model describing how Lorenz force acts on planar semi‐infinite electrode surfaces.^[10c]^ They found that introducing the magnetic field can reduce the thickness of the diffusion boundary layer. As shown in Figure 1e, the thickness is δ_d_ before application of the magnetic field. The magnetic field induces a tangential flow with the speed of *U*, leading to a hydrodynamic boundary layer with a thickness of δ_h_. Correspondingly, the thickness of the diffusion layer δ_d‐B_ is proportional to (*n*C*B)^−1/3^, where *n* is the number of electrons involved in the Faradaic process and C* is the bulk concentration of the active species. Therefore, the reduced thickness leads to an enhanced supply of active species increasing the mass‐transfer‐limited current.

For the convection caused by the Lorentz force, a magnetic electrode/catalyst is not necessarily required. The magnetic field is generally uniform without distortion, while the current density can be distorted due to the electrode's periphery or its unevenness. Nevertheless, the Lorentz force does not always dominate when a macroscopically uniform magnetic field is applied. When ferromagnetic/ferrimagnetic electrocatalysts are tested, the nonuniform magnetic field is generated locally and microscopically in the electrolyte. As a result, the induced magnetic field is located near the ferromagnetic species rather than on a long‐range scale. Additionally, when the ferromagnetic/ferrimagnetic component has been magnetized before the experiment, the Kelvin force can be generated without applying an external magnetic field since a considerable retentivity persists for a ferromagnetic/ferromagnetic material.

## 3 Magnetic Hyperthermia

Magnetic hyperthermia is proposed to facilitate electrocatalysis reactions. Magnetic hyperthermia is a phenomenon in which intense heat is generated in the vicinity of magnetic nanoparticles by an external high‐frequency alternating magnetic field (AMF). This strategy enables precise control of the operating temperature to improve different chemistries, such as water‐splitting efficiency.[^7b^, ^13^]

Electrocatalytic water splitting for the hydrogen evolution reaction (HER) is a promising and practical option because hydrogen can work as a versatile energy carrier but this electrocatalytic reaction faces the dilemma of low conversion efficiency and high cost. A grand challenge is the search forexcellent candidate electrocatalysts with low overpotential and a small Tafel slope to drive hydrogen evolution. Most research has been directed toward preparation strategies to achieve excellent HER performance, e.g., constructing various nanostructures and introducing vacancies. The magnetic field could play an essential role in circumventing scientific barriers in this context. The localized heating offered by AMF can significantly accelerate the reaction rate while avoiding unnecessary energy waste and ameliorating device degradation. This strategy has already been applied to biomedical treatment years ago.^[16]^

**Figure 2:**
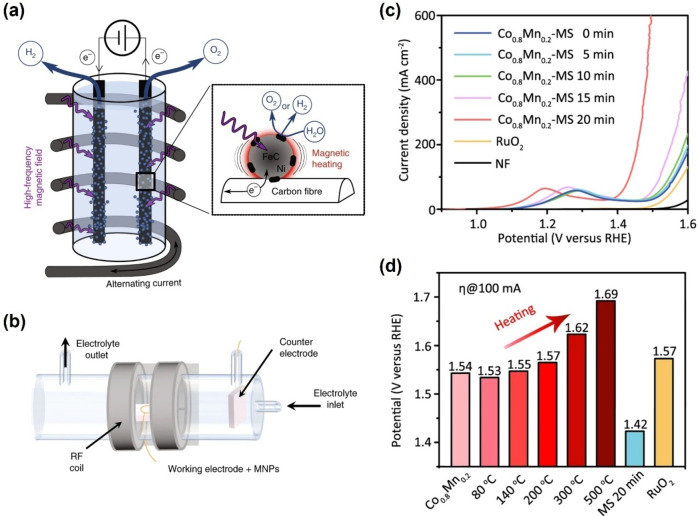
a) Schematic of the magnetic hyperthermia‐assisted water‐splitting process. b) Schematic of the experimental setup.^[17]^ Reproduced with permission. Copyright 2018, Springer Nature. c) Polarization curves of the Co_0.8_Mn_0.2_‐MOF with alternating magnetic stimulation; d) The onset potential (at 100 mA) for Co_*x*_Mn_1−*x*_‐MOF with different annealing temperatures and 20 min MS as reference.^[19]^ Reproduced with permission. Copyright 2021, The Authors.

In 2018, Niether et al. first applied high‐frequency AMF as a doping factor to facilitate electro‐driven water splitting in an alkaline water electrolyzer (AWE).^[17]^ The magnetic hyperthermia heating process and the experimental device are shown in Figure 2a and b, respectively. The authors used carbon‐felt‐supported core‐shell magnetic and catalytic nanoparticles (NPs) as electrode material. Under radiofrequency (300 kHz) AMF, the NPs magnetization changes direction with the AMF direction, and the induced work generates heat, which elevates the NPs temperature. In addition, the heating of the NPs, characterized by the specific absorption rate value,^[18]^ enhances the reaction kinetics. Under *B*=48 mT, *J*=18 mA cm^−2^, oxygen evolution reaction (OER) and HER chronopotentiometry measurements showed decreased overpotential by 250 and 150 mV, respectively. This enhancement of OER kinetics is equivalent to a ≈200 °C rise of the cell temperature. However, in practice, the temperature increased by only 5 °C. The significant result suggests that the current heat‐diffusion equations underestimate the surface local temperature of magnetically heated nanoparticles substantially. The localized heating does not only accelerate the reaction rate by the temperature effect but also reduces the consumption of energy and extends the service life of the electrolysis cell by avoiding heating up the whole electrolyzer. This work provides a new practical strategy to elevate the overall electrochemical performance in an energy‐efficient way.

Following these pioneering AMF investigations, recent work has continued elucidating the impact of magnetic hyperthermia on the catalytic performance using AMF. Zhou et al. synthesized Co_0.8_Mn_0.2_ metal–organic frameworks (MOFs) and applied AMF to form a thermal‐differentiated superlattice, and spin reconfigurations can be triggered by this magnetic stimulation (MS).^[19]^ Figure 2c shows that the OER activity increases with MS process time up to 20 minutes. It is known that conventional heat treatment may have a similar effect, but as Figure 2d shows, the MS‐processed sample exhibits superior performance. The structure transition can be easily relaxed after removing the heat sources in the heat treatment. Moreover, overheating can also lead to the collapse of the crystalline structure, which harms OER performance. This work provides a new insight for boosting OER performance in spin‐related electrocatalysts, inspiring a new strategy to regulate spin configuration and steer reaction kinetics.

## 4 Magnetic‐Field‐Driven Transport Phenomena

Adding magnetic components to electrochemical systems allows the magnetic field to play a significant role in mass transport and/or electron transfer, enhancing the overall efficiency of the process. This section discusses how magnetic fields affect the whole electrochemical system and the possible involved mechanisms.

High‐speed mass transport toward the electrode surface is highly desired for the enhanced performance of electrochemical devices. However, chemical conversion for most reactions is mainly limited by the slow diffusion of reactants and products toward or away from a reactive surface. Thus, increasing diffusion by forcing a convective flow without increasing energy consumption is essential. One promising approach to enhance the transport of species in the solution is applying a magnetic field to the system. This strategy requires understanding the effects of the magnetic field on electrochemical performance, including the effects of Lorentz and Kelvin forces. As an illustration, the analogy between magnetic fields and electrode rotation to generate convection has been proven previously.^[20]^ The equivalence of the magnetic field effect with that of a rotating disk electrode supports the idea that the field induces convection in the solution. This section covers examples showing the magnetic‐field‐driven transport effect. We review what has been achieved in magnetic‐field‐driven transport phenomena in the very recent past by focusing on the most promising developments. For each example, the effect‐dependent mechanisms behind the enhanced performance are discussed. Specifically, three ways of how magnetohydrodynamics (MHD) acts on electrochemical reactions are discussed, i.e., convection‐based bubble removal, convection‐modified mass transfer, and magnetic attraction. We highlight the roles of the Lorentz force and the Kelvin force in electrochemical reactions, point out the Kelvin force's significance in MHD, and describe their applications in electrodeposition, electrocatalysis, and batteries.

### 4.1 Magnetohydrodynamics (MHD)

#### 4.1.1 Convection‐Based Bubble Removal

This section introduces the reader to a growing body of interdisciplinary literature on the effects of magnetic‐field‐driven bubble removal on electrochemical systems’ energy and mass transfer efficiency.

The electrogenerated bubbles are a fundamental issue that influences energy and mass transfer in gas‐evolving electrodes, particularly during OER or HER. The targeted reduction or oxidation process is often coupled with a gas‐evolving redox process as a sacrificial reaction. The reaction on the active sites of the electrode surface results in supersaturation of the solution, which then accelerates the formation of gas bubbles. These bubbles then grow progressively over time until a critical size is achieved, at which they leave the electrode surface. From a practical point of view, bubbles attached to an electrode surface cover active sites during nucleation and growth in electrolytic cells. Therefore, they disturb the current distribution as sites become progressively occupied and freed during bubble lift‐off and isolate active sites from the reaction ions. Consequently, bubble generation at an electrode may be responsible for several undesired outcomes such as large reaction overpotentials and high ohmic resistances. The overpotential associated with bubble coverage has been described using a modified Tafel overpotential relationship wherein the current density is defined using bubble coverage. In addition to creating resistance, bubbles generated on the surface also lead to a decrease in concentration polarization and the number of active sites.^[21]^ Several strategies have been developed to remove bubbles, summarized in two categories. The first category requires an additional energy source to promote a change in bubble‐evolution behavior, such as the “self‐pumping” or hydrophobicity effect. In contrast, the second needs an external source for actuation, mainly based on flow, magnetic, and acoustic fields.

In 2006 Fukunaka et al. investigated the ohmic resistance of bubbles near Pt electrodes under microgravity.^[22]^ The ohmic drop, also called the IR drop, Δ*U*_ohm_, caused by the dynamic behavior of gas bubbles was measured using the current‐interrupt method.^[23]^ They influence the surface coverage at the electrode, *θ*, and the void fraction in the electrolyte, ϵ. In practice, the authors tested two kinds of platinum working electrode configurations for the HER process. One was a vertical cathode, while the other was a downward‐facing horizontal cathode‐over‐anode (C/A) setup. Using 0.1 N H_2_SO_4_ and 2 wt % KOH electrolytes, they found that the bubbles formed in the KOH medium were smaller than those formed in the H_2_SO_4_ medium. The different bubble phenomena in different electrolytes can be attributed to the difference in surface tension, and this may lead to different responses to the magnetic field. For the C/A setup, the bubbles coalesced and grew more considerably to a larger size on the electrode surface. The authors concluded that the ohmic drop caused by the bubbles would be mainly influenced by two factors: the bubble surface coverage, *θ*, and bulk void fraction, ϵ. The increasing bubble surface coverage can lead to a smaller effective electrode surface area. The bulk voidfraction, on the other hand, originates from the newly generated gas bubbles that are pushed into the bulk electrolyte and form a bubble froth layer; this layer equals tothe voids formed on the bulk conductive electrolyte. When the thickness of the bubble froth layer reaches a particular value, both *θ* and *ϵ* become constant, and the resistance increases linearly with the froth layer thickness.

To observe the effect of the magnetic field, applying an external magnetic field was proposed as one possible strategy to remove the bubbles and thus reduce the ohmic drop. By using platinum sheets as working and counter electrodes and a palladium microwire as a reference electrode, Fukunaka and co‐workers firstly studied the influence of electrode spacing.^[24]^ Surprisingly, they found that the degree of potential drop under the magnetic field was unrelated to the electrode spacing in both alkaline and acid medium. Meanwhile, they also found that the performance improvement was more evident in alkaline than in acid medium. These observations were related to the difference in the bubble detachment, which depends on replacing the electrolyte at the solid/solution interface, in other words, its wettability. The alkaline electrolyte shows much better wettability with the Pt metal surface than the acidic electrolyte. The result observed using an alkaline solution suggests that MHD convection can readily remove the bubbles from the electrode surface to reduce the void fraction and surface coverage. This result correlates well with previously observed IR diminution at a higher electrolyte velocity under a forced convection operation.^[25]^ The results also show that magnetic fields can facilitate both HER and OER performance, and the enhancement is more significant for OER than HER. The different gas bubble evolution dynamics may cause this difference. Even when an external magnetic field is not applied, buoyant hydrogen gas bubbles are easily removed without coalescence. Conversely, oxygen gas bubbles usually coalesce, grows to a more significant size, and stay at the working electrode for a longer time. Therefore, the magnetic‐field‐induced MHD convection can significantly remove oxygen bubbles before they coalesce.

**Figure 3:**
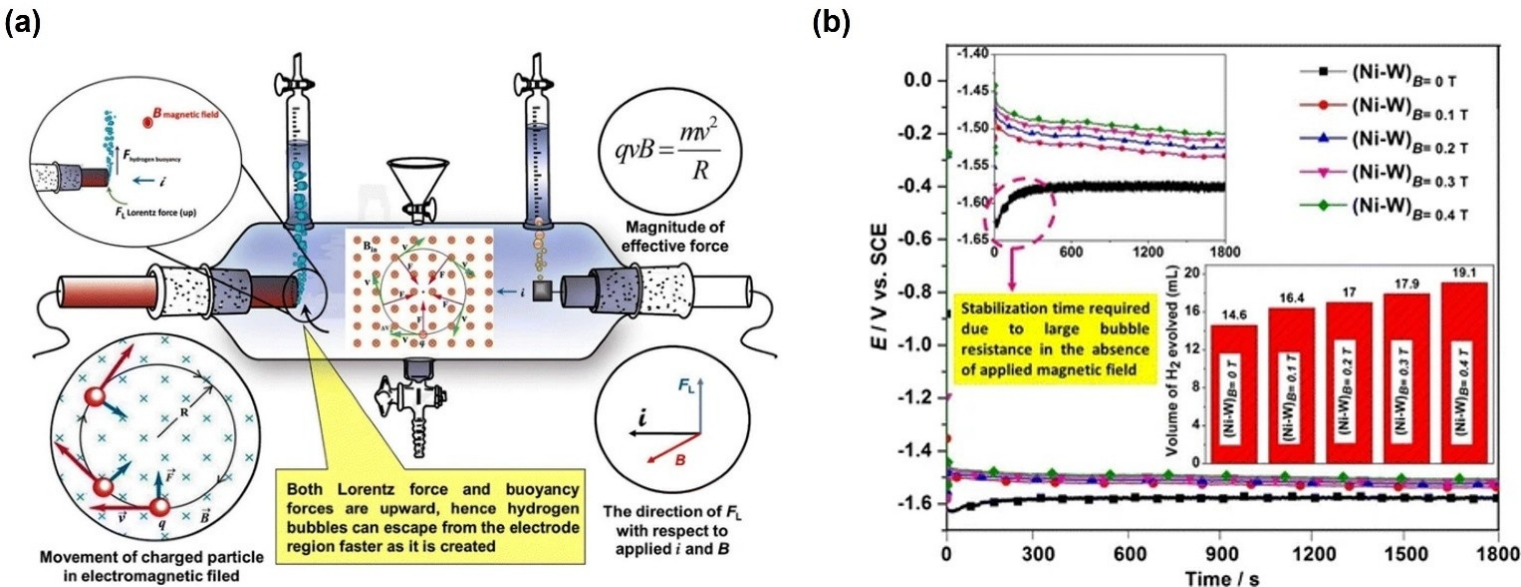
a) Schematic of magnetic‐field‐assisted HER by Lorentz‐force‐induced bubble removal; b) Chronopotentiometry curves and generated volume of hydrogen under different magnetic fields.^[26]^ Reproduced with permission. Copyright 2017, Springer Nature.

Elias et al. examined the effect of the magnetic field on the electrocatalytic activity of the Ni−W coated electrode.^[26]^ A remarkable difference is shown in the absence and presence of the magnetic field. Under an applied magnetic field, while the current density significantly increases, the overpotential decreases due to the bubble removal induced by buoyancy and the Lorentz‐force‐induced MHD convection (Figure 3a). The results indicated that electrocatalytic activity is enhanced by application of a magnetic field. When the magnetic field was increased from 0.1 T to 0.4 T, the produced hydrogen volumerose from 16.4 to 19.1 cm^3^ during 300 s. The enhanced catalytic activity is due to MHD power‐induced convection and rapid release of hydrogen bubbles. Similarly, Sambalova et al. also reported a 7 % enhancement in HER on a Pt electrode due to the change of the concentration gradient of hydroxide ions in the vicinity of the electrode surface. This observation has been supported by in situ probing of magneto‐optical properties and magnetocurrents.^[27]^

**Figure 4:**
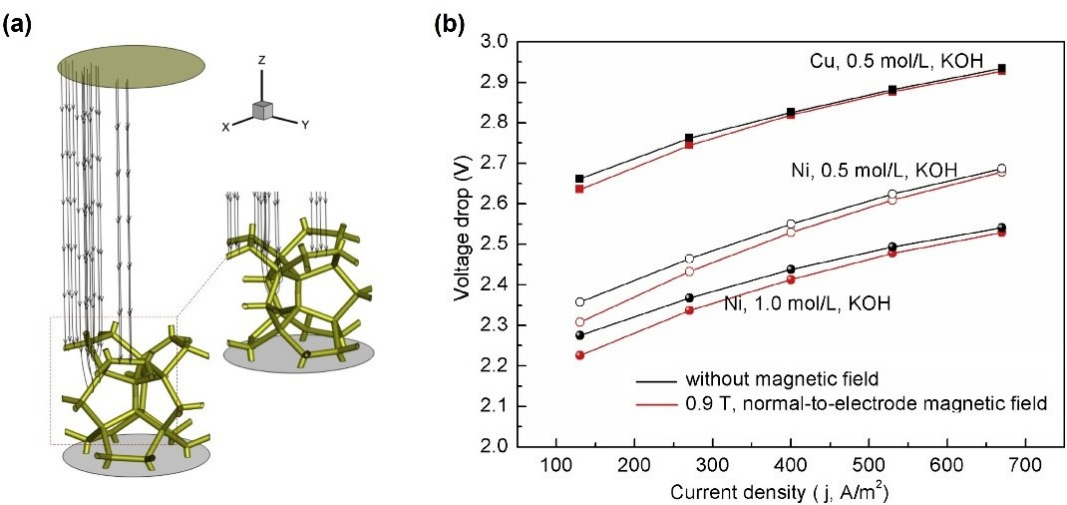
a) Schematic of the distorted current lines in the porous structure; b) the cell voltage drop versus current density.^[28]^ Reproduced with permission. Copyright 2019, Hydrogen Energy Publications LLC.

While all reports discussed in this section were performed using different catalysts or operating conditions, in a recent study the magnetic field was applied at different angles, vertical or parallel, to the surface. Macroscale MHD convection can only be generated when the magnetic field is not parallel to the current to generate Lorentz force. The external magnetic field is usually parallel to the electrode to get the best performance, so the current is vertical to the magnetic field. Therefore, macro‐convection is not generated when the external field is normal to the working electrode. However, the magnetic field may also facilitate water electrolysis even in this orientation for porous electrodes, as reported by Liu et al.^[28]^ By applying an electrode‐normal magnetic field to the cathodic working electrode, they found that the cell voltage can drop 2.5 % under a current density of 130 A m^−2^ when porous nickel is used, as shown in Figure 4b. Using the numerical simulation, they proposed that the porous structure of the electrode would distort the current and generate the Lorentz force that causes local MHD convection. A schematic of the distorted current is shown in Figure 4a. In their simulation, the magnetism of the working electrode was not considered, which means the Kelvin force caused by the magnetic gradient was not taken into account. Additionally, the same authors highlighted that the energy consumption was reduced by 3.4 % when a 0.9 T magnetic field was applied to foam electrodes in a 4.24 M KOH electrolyte.^[29]^

In essence, applying magnetic fields can accelerate bubble removal affecting the ohmic resistance and promoting the overall electrocatalysis performance. Both the Lorentz and Kelvin forces can play a role in this. The generation of Lorentz force does not require a ferromagnetic electrode, while this type of electrode can lead to higher efficiency given the induced Kelvin force. Meanwhile, the current distortion atthe edge or within the catalysts can also lead to extra convection even when the magnetic field is normal to the electrode.

#### 4.1.2 Convection‐Modified Mass Transfer

This section introduces the applications of convection‐modified mass transfer in electrodeposition, electrocatalysis, and batteries.

The most representative and well‐studied example of convection‐modified mass transfer in electrodeposition is that of copper, which was described decades ago.^[30]^ It has been proved that the MHD convection can accelerate the electrodeposition rate by reducing the diffusion layer thickness.^[30a]^ Initially, the study was mostly centered on theoretical analysis and calculation. However, only recently, with the advancement of characterization techniques, experimental observation has become possible.

**Figure 5:**
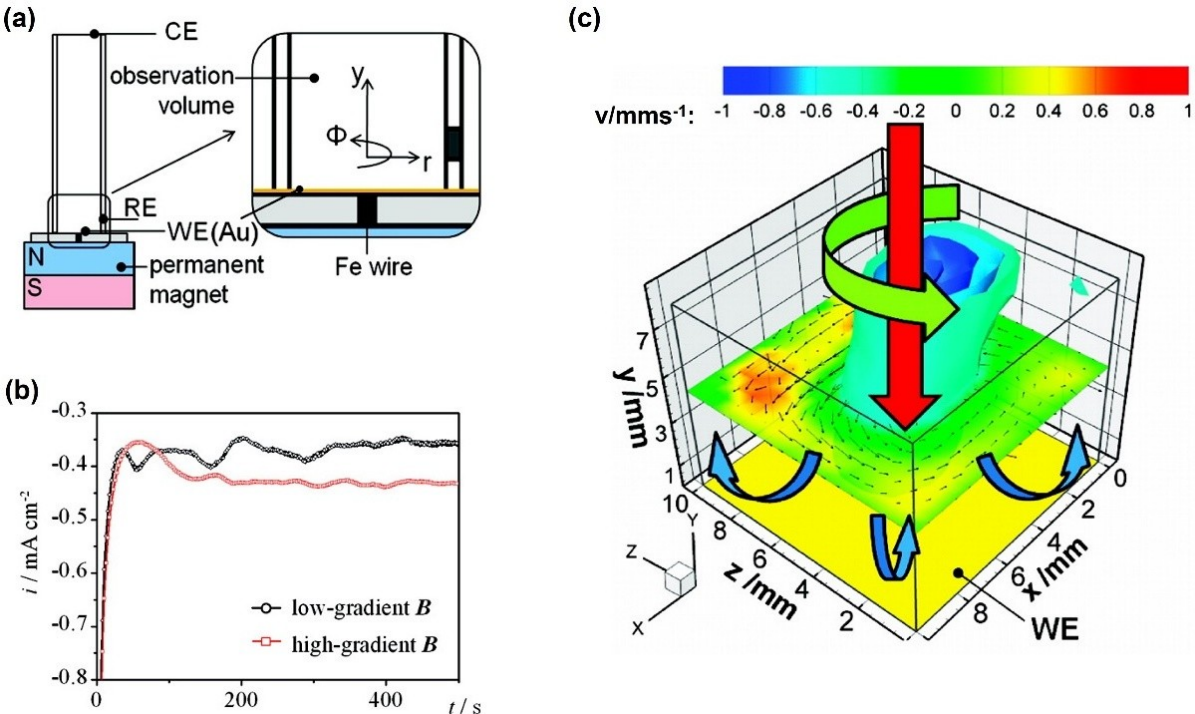
a) Schematic illustration of the experimental setup for the high magnetic field gradient experiments; b) Chronoamperometry curves for Cu deposition with low and high magnetic field gradient; c) Schematic of the velocity field under high magnetic field gradient. Reproduced with permission.^[20c]^ Copyright 2011, American Chemical Society.

The influence of a magnetic field on electrodeposition processes has long been known. This technique has recently been employed to deposit structured metal layers by the superposition of magnetic field gradients. In this regard, Tschulik et al. applied particle tracking velocimetry (PTV) to observe three‐dimensional electrolyte convection in situ for copper electrodeposition for the first time.^[20c]^ They used a gold‐covered glassy disk as the working electrode and Pt as the counter electrode. To address the influence of the magnetic gradient (∇B) effect, i.e., the Kelvin force, Fe wire was embedded in non‐conductive epoxy resin and placed at the middle of the top of the permanent magnet, as shown in Figure 5a. An empty epoxy resin was used instead for the control group with a low magnetic gradient. The potentiostat results for both low and high ∇B are shown in Figure 5b. Both low and high ∇B exhibit high current density at the first stage and then drop quickly, which indicates that the Cu^2+^ cations in the vicinity of the working electrode have been consumed, and the current is limited by the long‐term transfer of the Cu^2+^ cations. While the Lorentz force for both cases is similar, the Kelvin force comes into play. It introduces another convection that drives charged Cu^2+^ cations towards a higher magnetic field intensity, i.e., the working electrode in this case. It turns out that the current under high ∇B is 16 % higher than that under low ∇B. The three‐dimensional representation of the flow field high ∇B during electrodeposition is shown in Figure 5c. There are three velocity components: the circumferential velocity $u_{\phi }$ , the radical velocity $u_{r}$ , and electrode‐normal velocity *v*. A strong flow points to the center of the Fe wire, proving that the Kelvin force facilitates the Cu^2+^ diffusion. More recent work from the same group demonstrated that the Lorentz and the Kelvin forces affect each other, which induces additional convection.^[20a]^ They further pointed out that the Kelvin force persists longer than the Lorentz force and concluded that the Kelvin force is mainly responsible for the structuring effect. These observations inspired other research teams such as Coey and co‐workers and Gebert et al. to exploit this behavior to fabricate Cu deposition gradients.^[31]^

**Figure 6:**
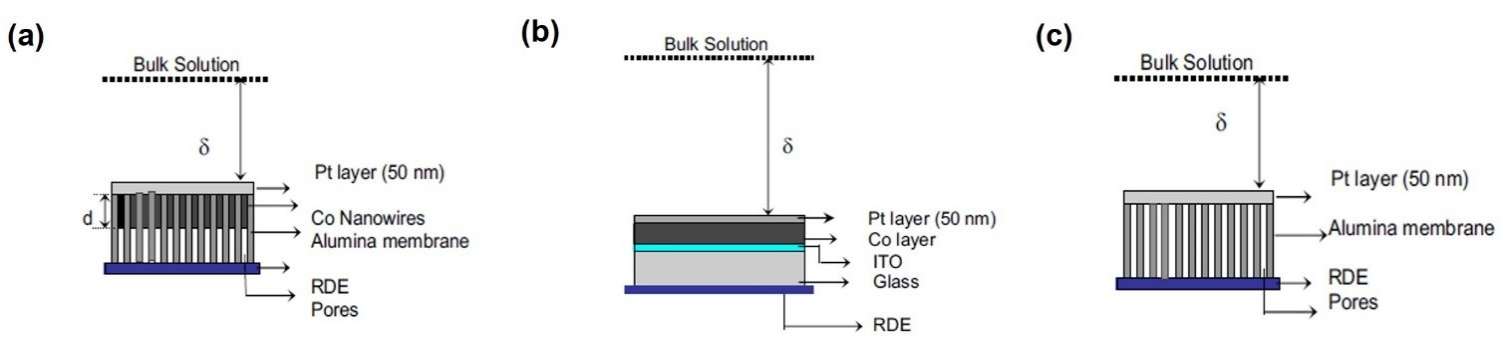
a–c) Schematic of three experimental setups, where δ is the thickness of the diffusion layer, where (a) can generate a gradient magnetic field; (b) generates a uniform magnetic field; and (c) does not generate a magnetic field.^[33]^ Reproduced with permission. Copyright 2009, (ECS) The Electrochemical Society.

In tandem with the studies focused on electrodeposition, strategies towards enhancing reaction rates for other electrochemical processes involving paramagnetic proprieties using appropriately designed electrodes that create a magnetic field gradient have also been developed. Due to the sluggish kinetics of the oxygen reduction reaction (ORR), this strategy at the oxygen electrode is an exciting example.^[32]^ From a molecular point of view, as the ground state oxygen molecules are triplet oxygen, i.e., containing two unpaired electrons, dioxygen is paramagnetic, and, like Cu^2+^ cations, will also be influenced by the magnetic field. To verify this hypothesis, Coey and his group used three different experimental setups to explore the role magnetic fields play in ORR.^[33]^ To study the effect of the Kelvin force, they employed and compared a Co nanowire to generate a high magnetic field gradient (Figure 6a) with a uniform Co layer (Figure 6b). Their chronoamperometry showed that under a 1.0 T magnetic field in an oxygenated borate bath under rotation, the enhancement of the Co nanowire setup (189 %) is almost one order of magnitude higher than that of the Co layer setup (31 %) and no‐Co setup (4 %) in a 0.0 T magnetic field. Their results highlighted the importance of the Kelvin force in magnetic‐field‐assisted ORR.

**Figure 7:**
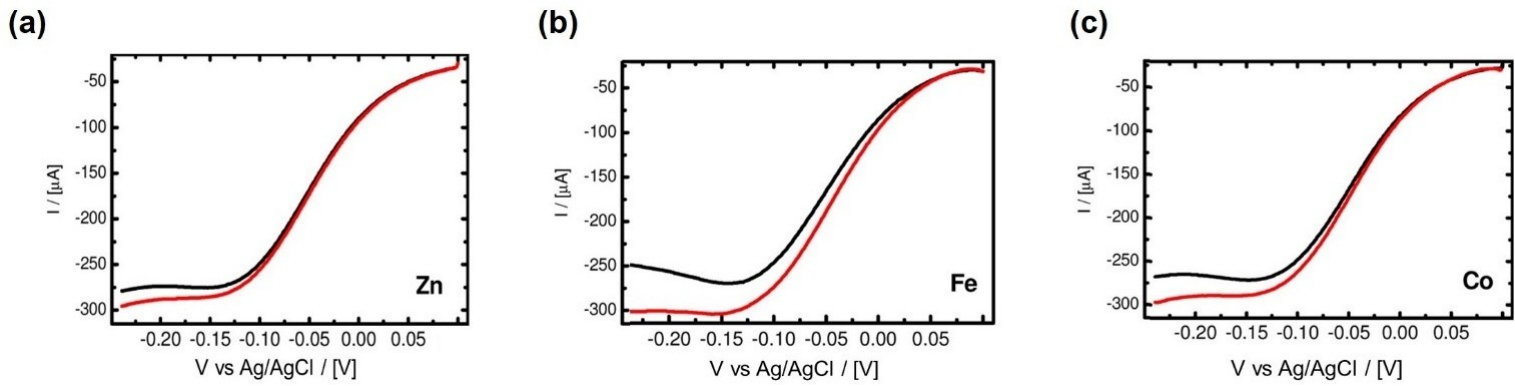
a–c) The cathodic scans for the ORR with (red) and without (black) magnetic field. a) Zn, b) Co, and c) Fe.^[34]^ Reproduced with permission. Copyright 2012, American Chemical Society.

**Figure 8:**
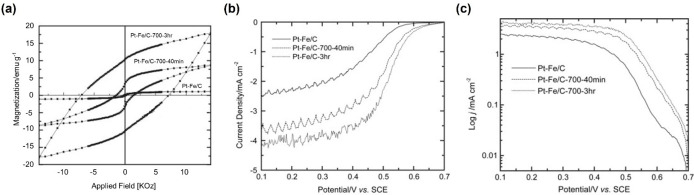
a) The hysteresis loop of three PtFe/C catalysts; b) Polarization curves of Pt−Fe/C samples for ORR; b) Mass‐transfer‐corrected Tafel plots for ORR.^[36]^ Reproduced with permission. Copyright 2009, Prof. T. Nejat Veziroglu.

They also embedded three metal particles (Zn, Co, and Fe) with similar shapes and sizes into electrode substrates to optimize the performance.^[34]^ They coated three metal particles with gold, Nafion, and Pt. The ORR tests were performed in oxygen‐saturated 0.5 M H_2_SO_4_ without rotation. Their maximum current increased to different extents when a 360 mT magnetic field was applied perpendicular to the electrode (≈3.0 % (Zn), ≈7.8 % (Co), and ≈11.6 % (Fe)) as shown in Figure 7a–c. The enhancement of Zn can be attributed to the Lorentz force. On the other hand, for Co and Fe, the generated Kelvin force can attract paramagnetic dioxygen molecules towards the electrode, which induces convection within the diffusion layer that accelerates the mass transfer. As Lee and his group reported, similar results can also be obtained from superparamagnetic ORR catalysts.^[35]^ They synthesized Ag core and Fe_3_O_4_ shell magnetoplasmonic nanoparticles (Ag@Fe_3_O_4_) with superparamagnetism. After application of a magnetic field up to 380 mT, 1.13‐fold high ORR efficiency was achieved. Most research on magnetic‐field‐facilitated mass transfer superimposes the magnetic field directly on the electrochemical systems. In this scenario, both the Lorentz and Kelvin forces are generated when the electrode is ferromagnetic; however, only Lorentz force will be produced when the electrode is nonmagnetic. For this reason, other research teams have directed their research to investigate the role of the Kelvin force solely. Zeng and co‐authors investigated three PtFe/C catalysts with different magnetic properties.^[36]^ As shown in Figure 8a, PtFe/C exhibits superparamagnetism while the samples PtFe/C‐700‐40 min and PtFe/C‐700‐3 h, which were annealed at 700 °C for 40 minutes and 3 hours, respectively, show ferromagnetism. The magnitudes of remanence, *B_r_*, are in the order of PtFe/C<PtFe/C‐700‐40 min<PtFe/C‐700‐3 h. After magnetization by a 1.4 T magnetic field, the ORR performance was tested in 0.5 M H_2_SO_4_ with a scan rate of 10 mV s^−1^ at room temperature without magnetic field. The linear polarization curves at 900 rpm are shown in Figure 8b. It can be seen that the ferromagnetic catalysts outperform the as‐prepared catalyst, and there is a positive correlation between the *B_r_* and ORR performance. The corresponding mass‐transfer‐corrected Tafel plots are shown in Figure 8c. In the kinetic region from 0.7–0.65 V, the Tafel slopes indicate that the magnetic field does not affect the kinetics. The current density difference was mainly due to the Kelvin force resulting in mass transfer facilitation. The magnetic gradient was generated at the interface of the ferromagnetic catalysts, which attracts the paramagnetic oxygen molecules and thus facilitates the mass transfer. This work proves that the Kelvin force can accelerate mass transport individually, reducing ORR's mass transfer limitations.

**Figure 9:**
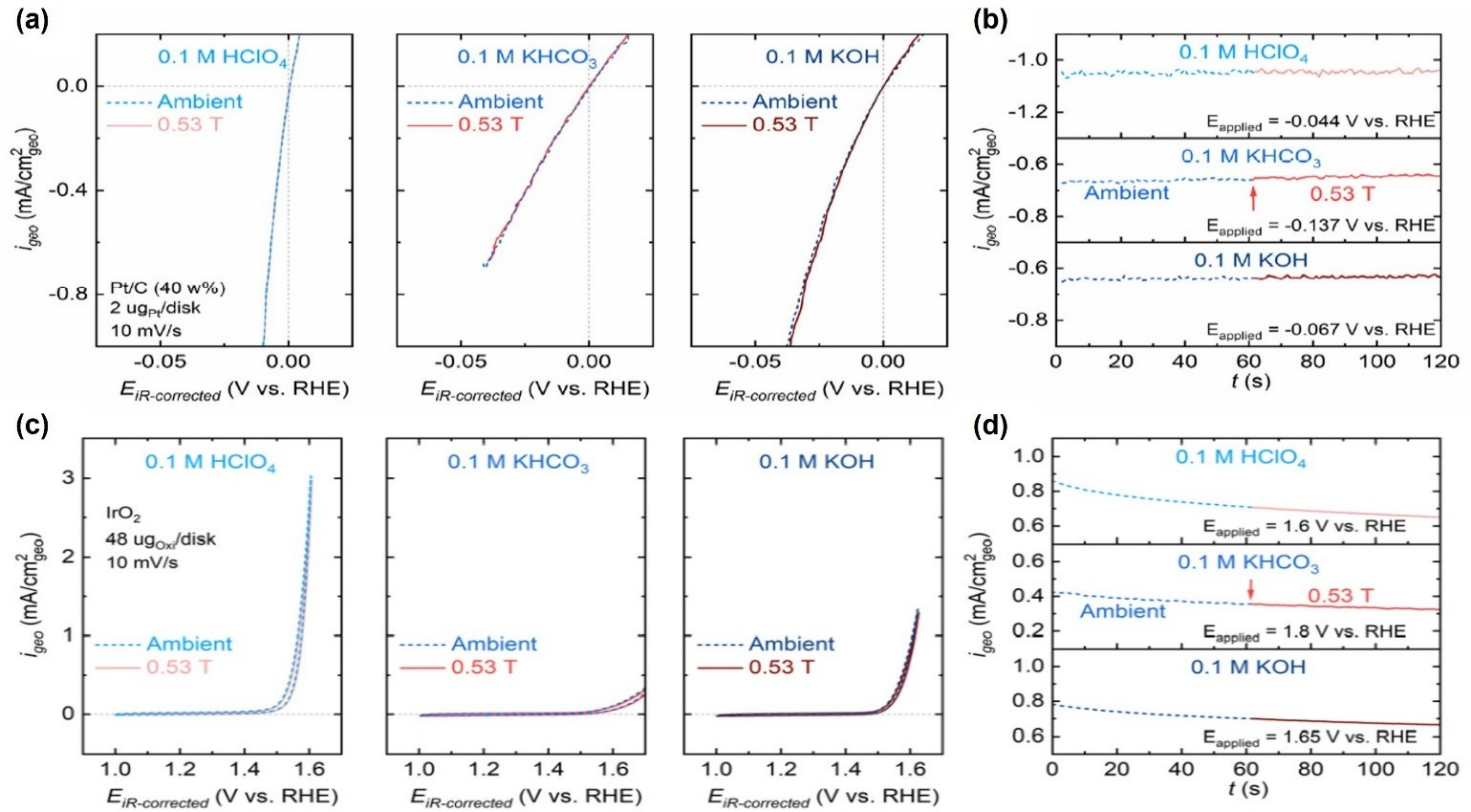
a, b) Electrochemical test results for a commercial Pt/C catalyst in HER with or without magnetic field. c, d) Electrochemical test results for a commercial IrO_2_ catalyst in OER with or without magnetic field. a, c) Positive‐going polarization curves. b, d) CA curves.^[15]^ Reproduced with permission. Copyright 2021 Dalian Institute of Chemical Physics, Chinese Academy of Sciences.

Based on the research above, the magnetic field can act on paramagnetic species and thus facilitate the reaction. According to this finding, our group applied a magnetic field to explore the possible reactive species involved in water splitting.^[15]^ Traditionally, the HER overall reactions have been written involving either acid protons or hydroxide ions in alkaline media. In acidic electrolyte, the reaction follows the Tafel/Volmer or Heyrovsky/Volmer mechanism.^[37]^ The Heyrovsky and the Volmer steps have conventionally been considered using hydroxides rather than protons as reactants in an alkaline medium.[^37a^, ^38^] The reaction pathways mentioned above in acid and alkaline media, where either H^+^ or H_2_O/OH^−^ would be the reaction species, have been used to explain the performance of Pt in acidic vs. alkaline electrolytes. However, so far, there is no concrete experimental evidence to support this assumption. In an attempt to explain the mechanistic reason, Xu et al. used a magnetic field to understand proton transport in HER and hydroxide ion transport in OER and their possible implications on both reactions.^[15]^ The authors applied a 0.53 T magnetic field parallel to the working electrode (vertical to the current) during the HER/OER process. Commercial Pt/C was used as a catalyst for HER in the preparation and tested in electrolytes with different pH values. If the reactant for HER in acid is a proton, a current change will be observed since it is a charged species, which Lorentz's force influences. Nevertheless, in Figure 9a,b, the current did not change with or without a magnetic field at any pH value. As shown in Figure 9c,d, similar results are also obtained for OER. These results imply that the magnetic field does not change the HER/OER performance. These unexpected outcomes can probably be attributed to the proton and hydroxide ion transport behaviors. Unlike charged species like Cu^2+^ ions mentioned above, proton and hydroxide transportation can be explained by Grotthuss theory.^[39]^ To reach the electrode surface, H^+^ and OH^−^ are transported via the hydrogen bonding between the water molecules. One H^+^ in the first water molecule can “jump” to the second molecule to form H_3_O^+^ and this process is repeated until a proton reaches the electrode surface. Hydroxide also follows a similar pathway. This mechanism highlights the continuous short‐distance jumping behavior of H^+^ and OH^−^ poles apart from long‐distance physical movement like that of Cu^2+^ cations. Using a magnetic field, the authors addressed the probability that H_2_O is the real reactant in water splitting rather than H^+^ and OH^−^.

As a sustainable energy storage solution, lithium‐ion batteries play a central role in the climate change roadmap without emission of greenhouse gases. They possess a favorable redox potential and efficiently store energy for extended periods in applications ranging from portable devices to electric vehicles. Despite the remarkable progress achieved in this area, further advancements in the properties of their components are highly needed. In the field of magnetoelectrochemistry, strategies similar to those used in electrodeposition and electrocatalysis were adapted to batteries. Many groups have been interested in the topic due to the relevance that magnetic fields could have for tuning various features of the battery components. One major obstacle to their practical usage is the uncontrollable dendrite growth due to the nonuniform Li^+^ concentration at the electrode surface, resulting in poor cycling efficiency and severe safety concerns.^[40]^

Different strategies have been applied to minimize the growth of dendrites to enhance stability and safety. As stated in previous sections, the Lorentz force may affect the charged particles when their trajectory intersects the magnetic induction lines. When Li^+^ ions plate on the protuberances of substrates in a magnetic field, the electric field line deflects and intersects with the magnetic field line, inducing solution convection due to the Lorentz force. As a result, the Li^+^ deposition sites are broadened, and the electrodeposition on the current collector is distributed homogeneously.^[41]^ Dendrite tips were thus greatly minimized due to the Lorentz force. Morphologically, Li tends to grow into uniform microspheres. Under a magnetic field, they grew a little bigger and became connected to form a flattened petal‐like morphology. Further increasing the magnetic flux intensity reversed the trend and turned the deposited Li back to microspheres. Interestingly, no dendrites were seen at low or high current densities.

**Figure 10:**
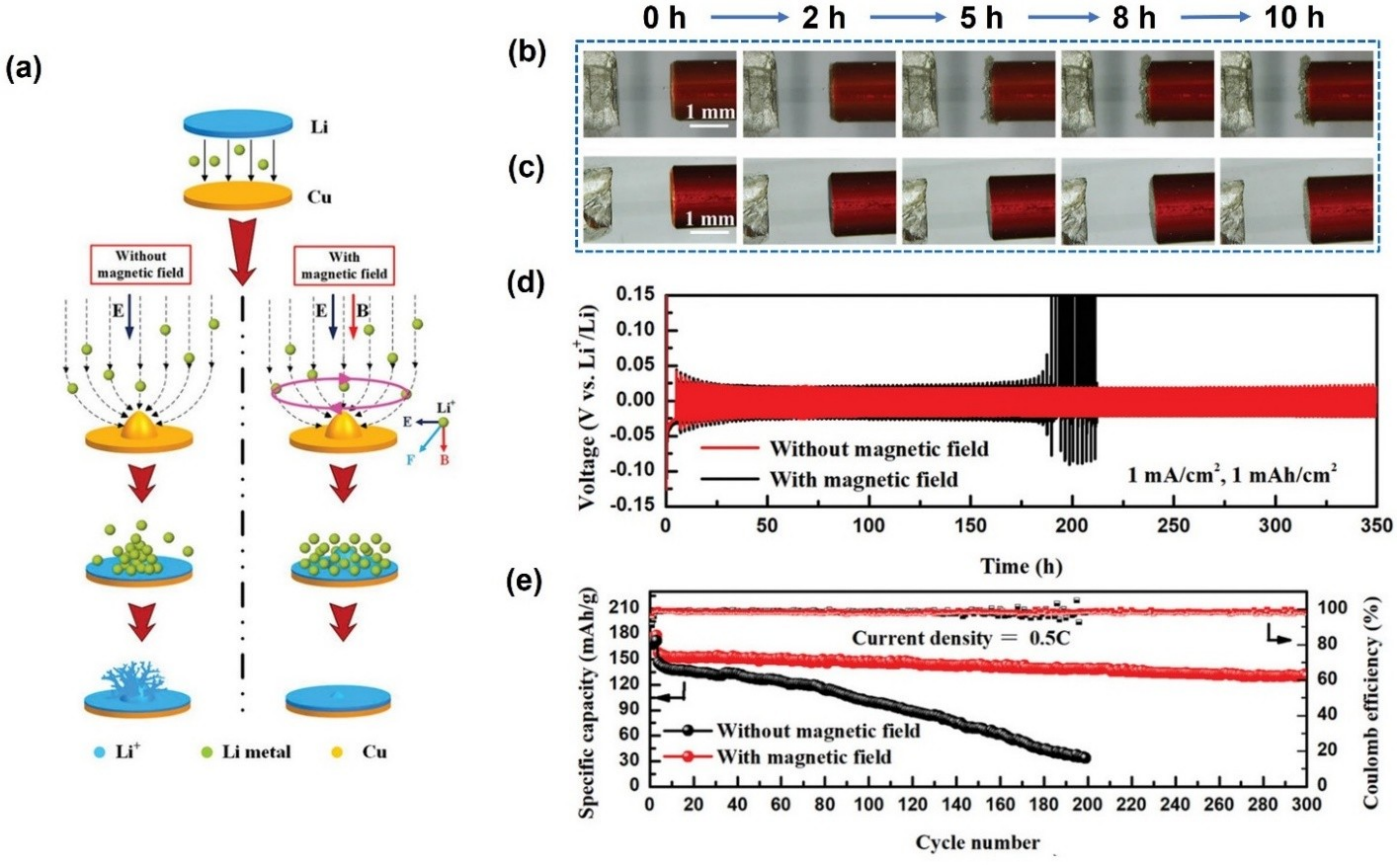
a) Schematic of Li^+^ deposition with or without magnetic field; b, c) Digital photos of Li deposits with (b) or without (c) magnetic field at different deposition times; d) Long‐term galvanostatic discharge/charge test with and without magnetic field; e) Cycling performance of full cells with and without magnetic field.^[42]^ Reproduced with permission. Copyright 2019 Wiley‐VCH.

Similarly, Shen et al. applied a magnetic field parallel to the electric field, as shown in the right panel of Figure 10a.^[42]^ A ferromagnetic NiCo alloy film was coated on a copper matrix to enhance the response to the magnetic field. In situ digital photos of Li deposition with and without magnetic fields are shown in Figure 10b, c. After 10 h deposition, no dendrite is observed for the Li layer with a thickness of about 40 μm under a magnetic field while a dendrite is formed, and the thickness is about 100 μm without a magnetic field. The authors explained that the Lorentz force acts on the charged Li^+^ cations, reducing the rate of protrusion growth by giving the Li^+^ cations a centripetal force. The cycling stability performances in symmetrical cells and full cells were also tested. The result shows that in the half cell (Figure 10d) and the full cell (Figure 10e), the cycle life and Colomb efficiency exhibit significant improvement under the magnetic field, confirming the positive effect of the magnetic field on lithium deposition.

It is worth mentioning that although many works report similar positive effects,[^41^, ^42^, ^43^] the magnetic field is not always beneficial to prevent dendrite growth. Experimental data supports that the MHD effect is conducive to eliminating dendrites, but some work has reached the opposite conclusion.

For example, Yu et al. pointed out that MHD can affect dendrite growth in three ways: uneven current distribution across the electrode, enhanced mass transport, and redistribution of Li^+^ ions around lithium dendrites.^[14]^ While the first effect accelerates the growth, the latter suppresses the growth. The applied magnetic field exacerbated the dendrite growth rather than suppressing it in their work. The current understanding of the MHD effect is not comprehensive, especially its influence on the behavior of Li^+^ ions at the electrode surface. Therefore, it is necessary to study the role of the MHD effect on the behavior of Li by designing different types of electrodes and corresponding numerical simulation analysis.

To summarize this section, the magnetic field application can facilitate the mass‐transfer‐limited electrochemical process. The Lorentz and Kelvin forces can be involved in the process, but when a magnetic field gradient exists, the Kelvin force plays a leading role compared to the Lorentz force.^[44]^ Those findings provide new strategies for electrode configuration design, especially when paramagnetic species are involved in the electrochemical reaction.

### 4.2 Magnetic Attraction

**Figure 11:**
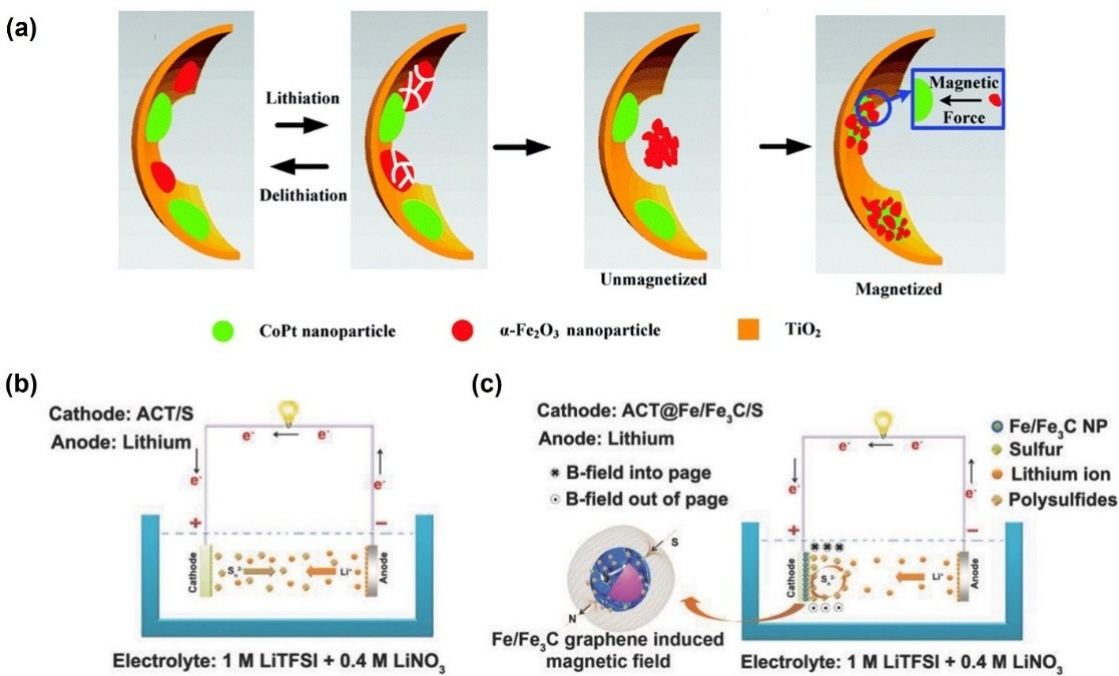
a) Schematic of reusing pulverized α‐Fe_2_O_3_ during the lithiation/delithiation process bythe internal magnetic field. Reproduced with permission.^[46]^ Copyright 2017 The Royal Society of Chemistry. b, c) Schematic of the polysulfide‐trapping mechanism before (b) and after (c) introduction of ferromagnetic Fe/Fe_3_C. Reproduced with permission.^[48]^ Copyright 2018 Wiley‐VCH.

The applications discussed in the previous sections mainly focus on the MHD‐facilitated the mass transfer, in which the Lorentz and Kelvin forces can accelerate the mass transport and modify the deposition structure. Nevertheless, the magnetic field can attract charged or paramagnetic species and thus limits their diffusion, which may also benefit batteries. Due to its high theoretical capacity, hematite (α‐Fe_2_O_3_) is a promising anode material candidate for lithium‐ion batteries (LIB). Unfortunately, its considerable volume change and aggregation during the lithiation/delithiation process lead to poor cycling stability. As a result, α‐Fe_2_O_3_ will usually be pulverized and detached from the electrode.^[45]^ If pulverization is inevitable, one possible strategy is to attract pulverized powders to the current collector. Considering the weak ferromagnetic properties of α‐Fe_2_O_3_ and the formation of metallic Fe during charge/discharge processes, Tang et al. fabricated a ferromagnetic three‐dimensional‐ordered macroporous TiO_2_/CoPt/ α‐Fe_2_O_3_ (3DOMTCF) composite in the presence of ferromagnetic CoPt nanoparticles to adsorb the paramagnetic Fe_2_O_3_ nanoparticles.^[46]^ As shown in Figure 11a, after magnetization, the reversible capacity of 3DOMTCF is enhanced, and the life cycle is extended. These results could be attributed to the internal magnetic field and its unique structure to reuse the pulverized Fe_2_O_3_.

Other approaches used for polysulfide inhibition include magnetic‐field‐enhanced polysulfide‐trapping mechanisms. For example, lithium–sulfur (Li−S) batteries suffer from fast capacity decay and poor cycling life originating from the dissolution/shuttling of lithium polysulfides.^[47]^ Gao et al. introduced magnetic nanoparticles with graphene shells onto a flexible cotton fiber as a cathode to trap polysulfide for better device performance.^[48]^ They embedded ferromagnetic iron/iron carbide with a graphene shell into the activated cotton textile (ACT@Fe/Fe_3_C/graphene) as a sulfur host and finally formed ACT@Fe/Fe_3_C/S as a cathode. The ferromagnetic Fe/Fe_3_C nanoparticles can generate a magnetic field around the cathode, which obtunds reduces the shuttle effect of polysulfide; as shown in Figure 11b, c, the magnetic field facilitates the formation of cathode/electrolyte interface, which blocks the dissolution of polysulfides. In other words, the magnetic field successfully traps the polysulfides in the vicinity of the cathode. Compared to pure ACT/S, ACT@Fe/Fe_3_C/S exhibits high initial discharge capacity, outstanding rate performance, and long lifespan. The plausible mechanism of polysulfide trapping was also provided.

In summary, the magnetic field can affect the battery performance in many ways. Besides the applications mentioned above, namely adjusting the mass transport (expediting or decelerating) by the magnetic force, an applied magnetic field can also affect electrode material preparation, battery status detection, and battery recycling. However, these other applications are beyond the scope of this review and have been discussed in Ref. [49].

## 5 Magnetic‐Field‐Enhanced Spin Selectivity

Apart from the physical acting forces and thermal effects, tuning electronic properties by applying an external magnetic field has become a new trend in magnetoelectrochemistry.^[8b]^ The latest research confirms that coupling the magnetic field with engineered electrocatalysts is a promising novel strategy to enhance electron transfer by lowering the energy barrier.^[50]^ However, magnetic field effects on both electron‐transfer kinetics and electrochemical equilibria have proved more controversial, partly due to the difficulty of eliminating indirect mass transport effects. Based on theoretical analysis, Gracia et al. have proposed that the quantum spin‐exchange interaction (QSEI) of ferromagnetic materials can lead to spin‐selective removal of electrons, which is beneficial to OER performance.^[51]^ However, although some researchers attempted to validate this hypothesis experimentally and theoretically,[^51a^, ^52^] the evidence obtained is still indirect.

Applying an external magnetic field can make the electron spin more aligned for ferromagnetic electrocatalysts. Although the electron spin in a ferromagnetic material is aligned even before magnetization, the overall magnetism is still not seen, which can be attributed to the domain structure of ferromagnets. The magnetic moment inside each domain is aligned in one direction, and that direction varies with the domain. The randomly distributed domains cancel the total magnetization. Consequently, a ferromagnetic material shows macroscopically zero magnetization. As a result of applying an external magnetic field, all the magnetic domains within the material move or rotate towards the magnetic field direction and yield a net magnetization.^[53]^ The enhanced magnetization is expected to contribute to a more substantial spin‐exchange effect, which leads to better activity. The magnetic field applied does not change the electronic configurations of the catalysts, e.g., flip the spin of electrons from up spin to down spin or convert the cation from a low‐spin state to a high‐spin state. The magnetic field intensities need to be extremely large (tens or even hundreds of Tesla) to modify the electronic spin configurations,^[54]^ which is inaccessible in most cases.

This section mainly addresses the spin modulation effect of the magnetic field on ferromagnetic electrocatalysts in electrocatalysis. Its applications in multiple electrocatalytic reactions are discussed. It is worth mentioning that the kinetic and diffusion effects may occur simultaneously; suggestions on the exclusion of interference are also offered to help researchers obtain solid conclusions.

### 5.1 The Applications of Magnetic‐Field‐Enhanced Spin Selectivity on Electrocatalytic Reactions

The pioneering work on the magnetic‐field‐facilitated spin selective effect on OER was developed by Galán‐Mascarós and his group.^[55]^ They tested a series of OER catalysts in alkaline media under a magnetic field. They found that ferromagnetic OER catalysts like NiZnFe_4_O_*x*_ showed a notable enhancement after application of a magnetic field, while non‐magnetic catalysts like IrO_2_ did not. This work inspired others to explore the magnetic field effect in electrocatalysis.

**Figure 12:**
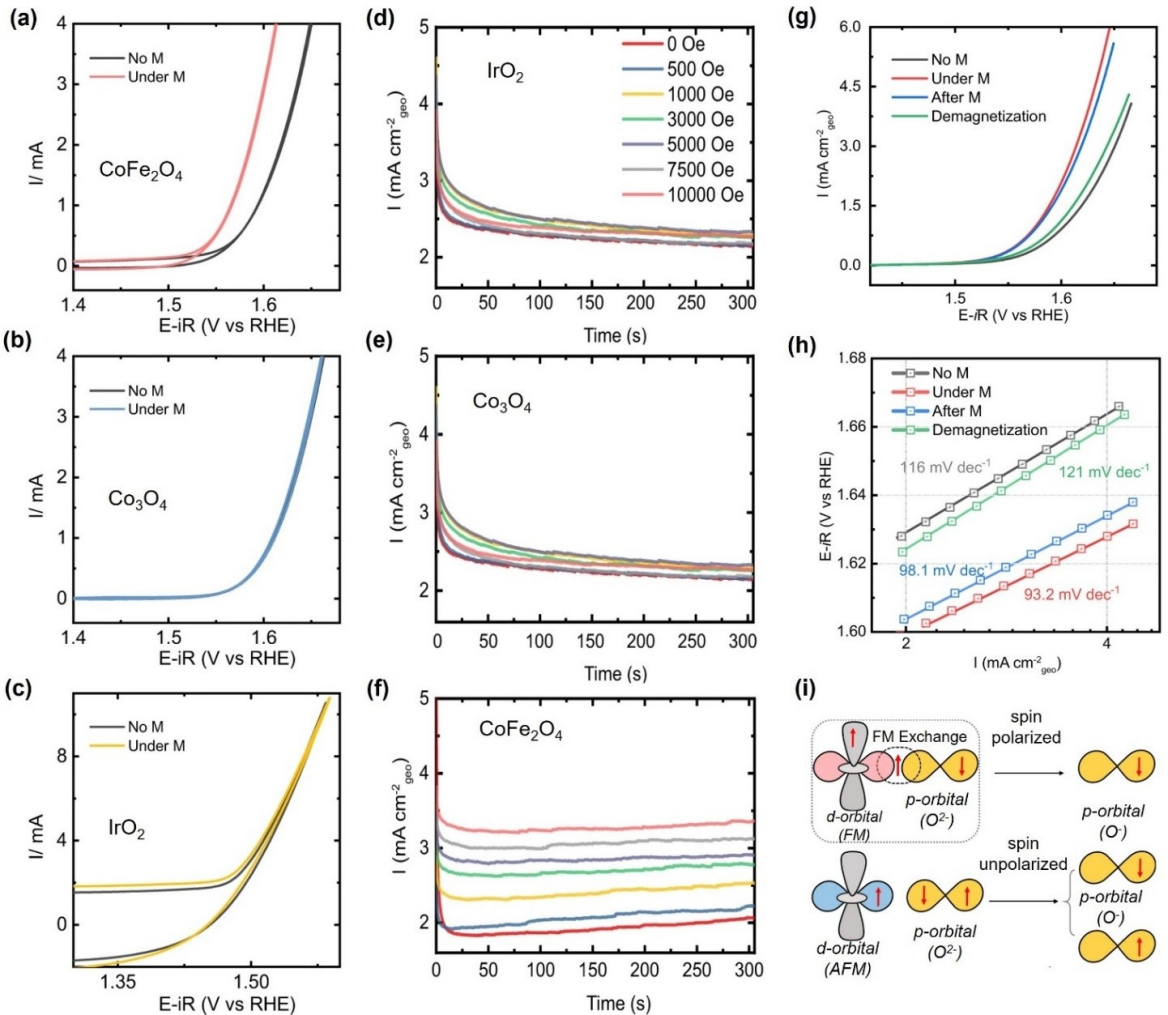
a–c) CV curves in 1 M KOH with and without magnetic field; d–f) CA curves under different magnetic fields in 1 M KOH; CoFe_2_O_4_ (a, f); Co_3_O_4_ (b, e); IrO_2_ (c, d); g, h) LSV curves and their corresponding Tafel plots of CoFe_2_O_4_ before the employment of magnetic field, application of magnetic field, removal of magnetic field, and after demagnetization; i) Scheme of spin‐exchange mechanism.^[56]^ Reproduced with permission. Copyright 2021 The Authors.

In 2021, Ren et al. compared three catalysts with different magnetic properties at room temperature: ferromagnetic CoFe_2_O_4_, antiferromagnetic Co_3_O_4_, and paramagnetic IrO_2_.^[56]^ A three‐electrode system was applied for the electrochemical tests, and 1 M KOH was used as electrolyte. The CV results and the corresponding Tafel plots in the presence and absence of 10 000 Oe magnetic field for the three catalysts are shown in Figure 12a–c. The OER performance of the ferromagnetic catalyst shows a significant promotion after application of a constant magnetic field, while no change was observed for non‐ferromagnetic catalysts. In addition, the Tafel slope of CoFe_2_O_4_ changed from 109 mV dec^−1^ to 87.8 mV dec^−1^, indicating the reaction rate‐determining step changed from the first step to a mixture of the first and the second steps. The setup for non‐ferromagnetic catalysts excludes the possibility of long‐term mass transportation and the magnetic effects on the electrochemical devices.

From a mechanistic point of view, if the mass transfer causes the increment, the observed current increment should also be seen with non‐ferromagnetic catalysts. Furthermore, the applied magnetic field can also act on the electrochemical cell because the cell components contain ferromagnetic metals. Under magnetic fields, those components can be attracted, thus changing the electrode distance and this may result in different electric resistance or even a short circuit. Similarly, CA tests were performed for IrO_2_, Co_3_O_4_, and CoFe_2_O_4_ under magnetic fields with different intensities (Figure 12d–f). The performance of CoFe_2_O_4_ shows an intensity‐dependence exclusively, further confirming the role of the spin selectivity. The increase in current using magnetized CoFe_2_O_4_ disappears after oscillation demagnetization, as shown in Figure 12g, h. After removal of the magnetic field, the promotion effect remains, attributed to *B_r_*’s remanence. Methanol oxidation reaction (MOR) and ethylene glycol oxidation reaction (EGOR) were also tested using CoFe_2_O_4_ as the catalyst under a constant magnetic field, and the results show no remarkable change after application of the magnetic field. The promotion is linked to spin modulation because the spin‐selective effect cannot exist when reactants, intermediates, and products are diamagnetic. All these results further confirm that the promotion originates from spin effects. The authors conclude that the ferromagnetism‐based spin‐dependent electron transfer would happen with a lower energy barrier, thus benefiting the reaction, as shown in Figure 12i.

**Figure 13:**
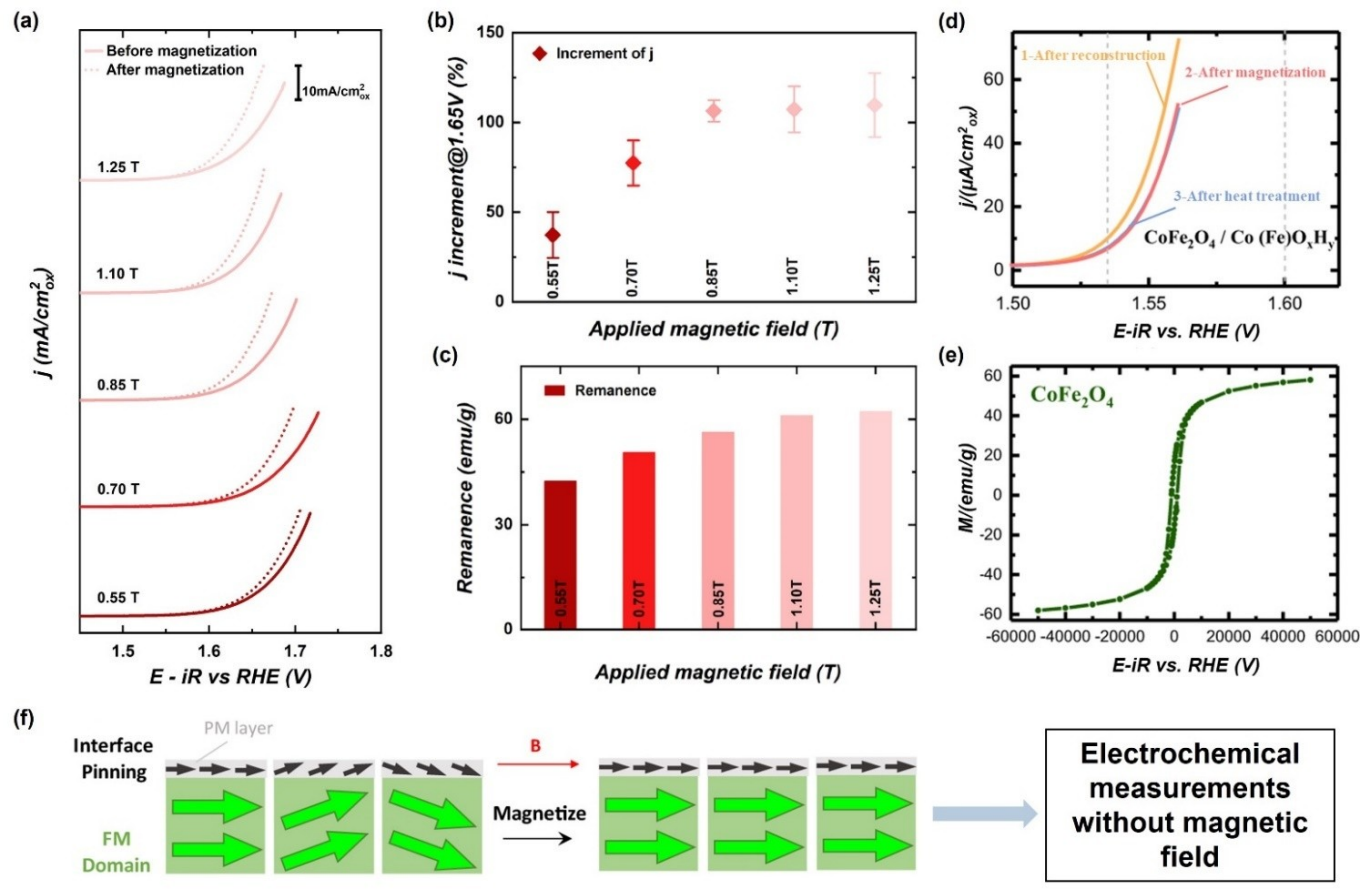
a) Polarization curves of SmCo_5_/CoO_*x*_H_*y*_ before and after magnetization; b) The relations between the current density increment and the magnetic field intensity applied for magnetization; c) The remanence *B_r_* of the SmCo_5_ core after being magnetized under different magnetic field strengths.^[57a]^ Reproduced with permission. Copyright 2021 Wiley‐VCH. d) Linear sweep voltammetry (LSV) of Co_3−*x*_Fe_*x*_O_4_(s) following the procedures: 1. after reconstruction (light blue curve), 2. after magnetization under 0.5 T for 15 min, and the removal of the magnetic field (yellow curve), and 3. after the post‐treatment at 120 °C for 1 min (pinkish curve); e) Magnetic hysteresis loops of CoFe_2_O_4_ oxide substrate; f) Schematic of the experiment procedure and the spin‐pinning effect at the interface between ferromagnetic (FM) magnetic domains and the thin paramagnetic (PM) oxyhydroxide layer. Note that the electrochemical measurements are conducted without magnetic field after magnetization of the working electrodes (catalysts).^[57b]^ Reproduced with permission. Copyright 2021 The Authors.

It should be noted that application of an external magnetic field on ferromagnetic materials leads to the magnetization of the materials, and the magnetized status can persist for a period. Thus, performing electrochemical measurements after magnetization of ferromagnetic catalysts also gives a similar result to what can be observed with an external magnetic field. For example, pre‐magnetization of SmCo_5_/CoOOH and CoFe_2_O_4_/Co(Fe)OOH can enhance the OER performance, in which the magnetization statues of ferromagnetic substrates persist after the pre‐magnetization treatment (Figure 13).^[57]^ The enhancement is achieved through the spin‐pinning effect. Conducting the electrochemical measurement without an external magnetic field using these pre‐magnetized ferromagnetic catalysts further confirms the role of spin in promoting OER, and other factors under the magnetic field can be completely excluded.

The studies, as mentioned earlier, mainly used catalysts to act as spin filters to realize spin selectivity. At the same time, the radical pair spin can also be modulated by the external magnetic field.^[58]^

**Figure 14:**
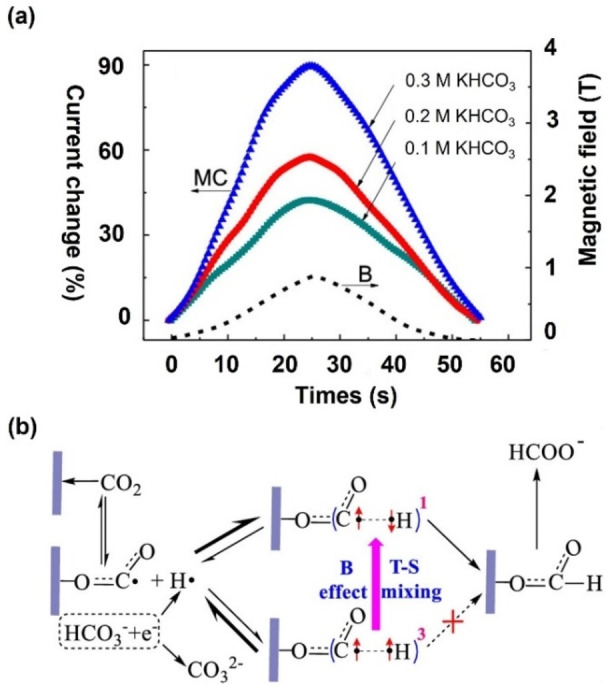
a) The current change versus time and magnetic field; b) The schematic of the proposed mechanism of the spin‐sensitive CO_2_ reduction reaction.^[59]^ Reproduced with permission. Copyright 2019 American Chemical Society.

In their report published recently, Pan et al. highlighted that the conversion rate of the electrocatalytic reduction of CO_2_ to formic acid using a nanoparticle tin electrode could be nearly doubled under a magnetic field compared to without a magnetic field.^[59]^ This result has been rationalized as a change in the efficiency of interconversion of the electronic singlet and triplet states of a radical pair [CO_2_^−.^⋅⋅⋅H⋅] originating from electroreduction of CO_2_ and bicarbonate (HCO_3_^−^). Figure 14b details the proposed reaction scheme. Statistically, the radical pairs are formed in a 3 : 1 ratio of triplet and singlet spin states. Pauli exclusion rules allow only singlet pairs to combine, forming the diamagnetic formate ion. Figure 14a shows the change in the catalytic current for CO_2_ reduction recorded by increasing the intensity of an applied magnetic field. Under 900 mT magnetic field, increasing the CO_2_‐saturated KHCO_3_ solution concentration from 0.1 M to 0.3 M is accompanied by an enhancement of peak currents from 42 % to 90 %, corresponding to an increase in the yield of formic acid from 40 % to 100 %. Similarly, the group also applied the same strategy to oxalate oxidation and obtained a 30 % current increment.^[60]^ The magnetic field effect seems to saturate at a high field, typical of radical pair reactions. The enhancement in both the current and the conversion rate is in line with a field‐induced increase in the conversion efficiency of triplet pairs into singlets, which react to give formate. The authors concluded that the magnetic sensitivity was observed because of the presence of two mechanisms for singlet–triplet interconversion: 1) the Δg mechanism, resulting from the difference in the electron Zeeman interactions of the two radicals, and 2) the hyperfine mechanism, arising from the magnetic coupling of the electron and the nucleus in H⋅. Since the former is expected to become more efficient and the latter less efficient, at high field, the authors argue that the former must be dominant.

So far, the spin modulation by the magnetic field has been applied to OER,[^55^, ^56^, ^57^, ^61^] ORR,[^61c^, ^62^] HER,^[63]^ CO_2_ reduction reaction (CO_2_RR),^[59]^ and oxalate oxidation reaction.^[60]^ From our discussion, the spin‐selective effect of ferromagnetic has been preliminarily shown as proof of concept. Thus, this emerging boosting strategy gradually shows its attractive potential in electrocatalysis. However, the magnetic field influences electrochemical reactions in many ways. Therefore, it is very challenging to ascribe the observed enhancement to spin modulation. The following section discusses the rationale for experimental confirmation of spin‐originated enhancement.

### 5.2 Suggestions on Interference Exclusion

As previously stated in this review, external magnetic fields can act on the whole electrochemical system on many levels. Therefore, rational experiment design must exclude interferences to investigate the real reason behind the boosted performance. Both MHD and electron spin modulation can simultaneously occur when the magnetic field is applied for mass‐transfer‐limited electrocatalytic reactions like ORR. The biggest obstacle is the rational control group setup. Ideally, this hypothesis should be tested on two samples with the same composition and structure but with different magnetism (i.e., one is ferromagnetic, the other is nonferromagnetic). To the best of our knowledge, it is impossible to perform these kinds of control experiments since different magnetism must systematically be accompanied by a change in compositions, structures, or temperature. In this case, an observed difference in performance would be tricky to interpret, and rational conclusions cannot be drawn.

Several possible interferences with spin‐originated performance enhancement might coexist. These interference effects could be observed as a result of the presence of three mechanisms: 1) the magnetoresistance effect that may affect the resistance of the catalysts,^[64]^ 2) MHD that could accelerate the mass transfer process and facilitate bubble removal, and 3) the changing magnetic field strength that may induce voltage based on electromagnetic induction.^[65]^

Different electrocatalytic reactions and different catalysts could have various responses to those phenomena. For example, the magnetoresistance effect may not have significant variations for powder catalysts regardless of their conductivities, as evidenced by the methanol and ethylene glycol oxidation reactions under the magnetic field.^[56]^ That is because powder catalysts are usually mixed with conductive carbonaceous materials. Therefore, the resulting conductivity of the powder will not limit the reaction performance. On the other hand, solid thin‐film electrodes made of low‐conductivity materials can probably give some quantifiable effect, which has not yet been confirmed by experiment.

For mass transport, it may have different effects on different reactions. As discussed above, magnetic fields can act on paramagnetic and charged species. Thus there is an observable difference in mass‐transfer‐limited reactions like ORR, but reactions like OER will not be affected by mass transfer, as evidenced by IrO_2_ across the pH range.^[15]^

For electrodes with a flat surface, the direction of the applied magnetic field MHD may still affect the system and be influential because the MHD effect originates from the Lorentz force, which varies along with the angle between the movement of the charged species and the applied field. For non‐flat surfaces, the acceleration effect cannot be eliminated simply by adjusting the current and magnetic field angle because the unevenness of the electrode can still distort the current, which can induce Lorentz force. Even for an atomically flat electrode surface, the edge may still distort the current to some extent. The Lorentz and Kelvin forces are ubiquitous in electrochemical systems but with different magnitudes.

In addition, MHD‐originated bubble removal may reduce the impedance of the reaction following two pathways. As discussed in Section 4.1.1, bubble formation can increase the ohmic resistance. If the bubbles are adsorbed to the catalyst, they may also block the active sites, thus impeding the reactions. For gas evolution reactions like OER and HER, the ohmic resistance can appreciably impact the performance only when the current is relatively high. While for all catalytic reactions, the bubble blockage will impair the catalytic process.

For electromagnetic induction, the change of magnetic flux intensity can induce extra potential. Still, if the magnetic field is not AMF, the induced bias is transient. Therefore, it will not affect the reaction, as evidenced by IrO_2_ and Pt/C in OER and HER, respectively.^[15]^

From the perspective of experiments, it is recommended to clearly describe the experimental setup for mass‐transfer limited reactions like ORR, including the magnetic field direction. For non‐mass‐transfer‐limited reactions like OER and HER, the angle between the magnetic field and the current will not affect the catalytic performance. The magnetic field may also affect the performance via altering ohmic resistance through the magnetoresistance phenomenon or the influence on the cell components. The magnetoresistance phenomenon may change the catalysts’ resistance, thus influencing the overall catalytic performance.^[66]^ However, it should not affect powder catalysts substantially because conductive carbon is usually added to the catalyst ink to conduct electrons.

The magnetoresistance effect can significantly influence the performance of solid thin‐film catalysts. Therefore, resistance measurements are desired before and during the magnetic field application in this case.

Practically, the design of control groups is crucial to avoid many unexpected interferences. Furthermore, commercially available catalysts (e.g., Pt/C, IrO_2_,) can exclude interference like mass transfer and experimental factors like devices. More specifically, as discussed in Section 5.1, the components of electrochemical cells may contain magnetic metals and may be attracted to magnetic fields, leading to resistance variation. Therefore, the fixation of the electrode position is of great importance. Meanwhile, using the targeted catalyst in non‐spin‐sensitive reactions like MOR would exclude the possible contribution from ohmic resistance. Both control group setups can help exclude some interferences, but they all have limitations. Specifically, commercial catalysts are usually nonmagnetic, and cannot generate magnetic gradients and Kelvin force. Although mass transfer will not affect reactions like OER and HER, it still plays a significant role in mass‐transfer‐limited reactions like ORR, especially when the promotion is not substantial enough. Using non‐spin‐sensitive reactions as a control experiment cannot exclude the influence of mass transfer because of different reactant species. Based on the analysis above, the experimental setup shown in Figures 5a and 6a coupled with commercial catalysts may be the ideal control group to exclude possible interferences.

## 6 Other Effects

**Figure 15:**
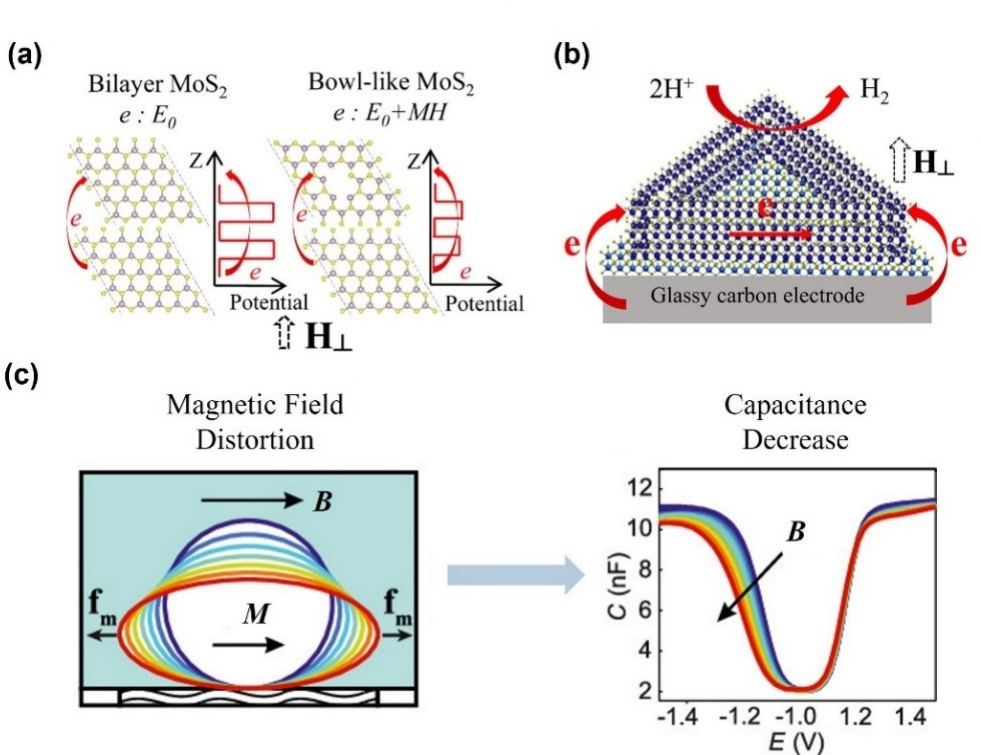
a) Schematic of electron transfer in the bilayer and bowl‐like MoS_2_ under an external magnetic field; b) Schematic of electron transfer in bowl‐like MoS_2_ during HER under an external magnetic field.^[67]^ Reproduced with permission. Copyright 2020 American Chemical Society. c) Schematic of the distortion and capacitance decrease caused by the Maxwell stress.^[68]^ Reproduced with permission. Copyright 2019 American Chemical Society.

Over the past few years, research in the areas discussed in previous sections has been dominant due to a push toward developing efficient applications. However, several groups reported the effect of the magnetic field on the electron transmission process. Zhou et al. reported a different reinforcement mechanism of HER.^[67]^ Using modified stepwise chemical vapor deposition, they prepared a ferromagnetic bowl‐like MoS_2_ electrode . They performed a comparative study using two different samples, the ferromagnetic bowl‐like MoS_2_ and bilayer MoS_2_. Based on the first‐principle density functional theory, they claimed that bilayer MoS_2_ is nonmagnetic while bowl‐like MoS_2_ is ferromagnetic. They reported a significant decrease in HER overpotential when a vertical magnetic field of 0.8 T was applied. Zhou et al. argued that the potential mechanism for such activity improvement should be assigned to the elevation in electron energy. As shown in Figure 15a, b, the electrons can hop from the conductive substrate to the active sites, promoting the electron‐hopping efficiency of the interlayer.

The magnetic field also plays a positive role in modulating the electrochemical double layer (EDL). Recent work by Dunne et al. in 2019 described the importance of magnetoelectrochemistry and its effect on the electrochemical double layer.^[68]^ They reported that the 0.5 T magnetic field could attract and distort the paramagnetic species on the electrode surface and contract the average outer Helmholz plane closer to the electrode by up to 0.25 nm as a result of the Maxwell stress.^[68]^ The double‐layer capacitance and charge‐transfer resistance were also decreased simultaneously, as shown in Figure 15c. While analyzing the intrinsic activity of the catalysts, the electrochemical active surface area (ECSA) is a commonly used parameter that is directly correlated with the double‐layer capacitance;^[69]^ therefore, it is recommended to measure ECSA before and after magnetization to check whether the Maxwell stress plays a role in the activity boosting. Sambalova et al. reported that when the magnetic field affects the EDL, it will simultaneously change the local concentration gradient of OH^−^ in the vicinity of the electrode surface plane and automatically change the observed current response.^[27]^

## 7 Summary and Outlook

It has been decades since the first use of applied magnetic fields in electrochemistry, and the research directions to find new applications for this method are gaining momentum. However, despite the considerable amount of data reported, it is still unclear whether magnetoelectrochemistry can be used as a commercial boosting method or only as an experimental tool to investigate different events and mechanisms at the electrode–solution interface. Catalyst engineering and novel electrode architectures have addressed some technological applications of magnetoelectrochemistry, while other complex applications, in which analysis and conclusions are weakened by constraints associated with multiple interferences, are still under debate. Uncertainty is the main issue due to the experimental control setup's ability to cope with the physical and structural parameter variability under magnetic field over time. In addition, there are many ideas and hypotheses to be explored in this area.

### 7.1 New Design Strategy for Electrode Preparation Based on the Magnetic Field

The future significant impact in this field would be the design of a robust experimental design enabling real‐time control by monitoring the variability of different parameters. Designing and coupling characterization methods as control setups is a sensible strategy for understanding mechanisms in scenarios of various applications. Catalysts with different magnetic properties like ferromagnetism, ferrimagnetism, antiferromagnetism, and superparamagnetism should also focus on their quantum spin‐exchange interaction.

It is known that many catalysts are diamagnetic; even ferromagnetic catalysts can probably undergo surface reconstruction and form a nonmagnetic surface.^[70]^ Since electrocatalysis is a surface‐determined process,^[71]^ those features significantly impede the application of the magnetic boosting strategy. One practical solution is using a spin pinning effect to construct a composite structure like a core‐shell structure.^[57b]^ The ferromagnetic/nonferromagnetic interface can still act as a spin filter and take effect as long as the shell is thin enough.^[61b]^

### 7.2 Expanding the Application Range and Exploring New Boosting Mechanisms

Although magnetic fields have been applied to various electrocatalytic reactions, there are still vast unexplored areas awaiting investigation, such as the nitrogen reduction reaction, nitrogen oxidation reaction, hydrogen oxidation reaction, and even electrocatalytic synthesis of organic molecules.

**Figure 16:**
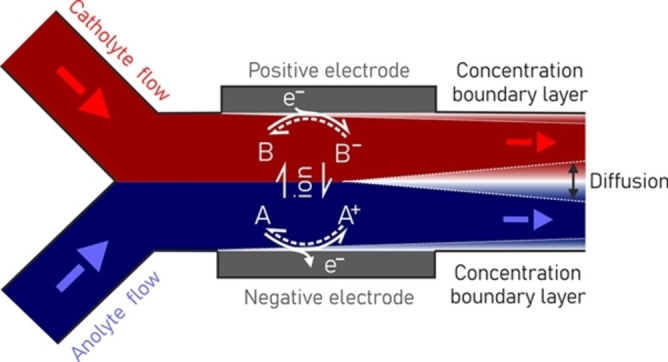
Schematic of a representative microfluidic flow cell.^[72]^ Copyright 2022 American Chemical Society.

The magnetic field may also be a potential manipulation method for devices like microfluidic flow cells. Microfluidics is the science which studies the behavior of fluids through microchannels and the technique of manipulating fluids in small conduits having at least one submillimeter dimension.^[72]^ In electrochemistry, microfluidics may also take advantage of precise flow control, targeted species directional transportation, and optimal thermodynamic equilibrium.[^72^, ^73^] A simple representative schematic of a microfluidic flow cell is shown in Figure 16. The flow regime in the microfluidic reactor system is primarily laminar, meaning convective mass transport is absent. Thus, mass transport is controlled mainly by diffusion and migration phenomena. Diffusion is the movement of species due to a concentration gradient resulting from substrate consumption at the electrode. Migration is the movement of charged species under a potential field.^[74]^ However, if the substrate concentration is low compared to that of other charged species present at the electrolyte, the contribution of migration to the mass transport can be neglected. Thus, mass transport is primarily dominated by diffusion in electrochemical microreactors. While only diffusion can be sufficient for a fluidic reactor using small channels due to the short diffusion distance to the electrodes, batch reactors with larger interelectrode gaps would require intense stirring to assist the mass transfer from the bulk to the electrode surface. In this scenario, the magnetic field could potentially manipulate the hydrodynamic proprieties of microfluidic devices. The application of magnetic field could be a promising strategy for introducing and enhancing convective mass transport. Coupling a magnetic field with microfluidic systems will be of great added value to enhance the performance of electrochemical microfluidic systems.

Other magnetic phenomena may also influence the electrochemical reactions and deserve further attention, such as the room‐temperature magnetoresistance effect,^[75]^ the Hall effect,^[76]^ and magnetostriction.^[77]^ As the term indicates, the magnetoresistance field means the electrical resistance can be changed when the magnetic field is applied.^[64]^ Since charge transport is crucial for electrochemical reactions, it can be expected that magnetoresistance can play an essential role in changing critical parameters for an electrochemical process. (Note that this influence is not necessarily desired.) While colossal magnetoresistance will lower resistance in response to a magnetic field, the positive magnetoresistance effect might increase the resistivity, which would be detrimental to the reaction.^[78]^

We believe that researchers should consider engineering aspects when designing a better test setup in this field. Adopting engineering strategies associated with research efforts will help advance the fundamental understanding of the complex processes occurring at electrode interfaces. Furthermore, translating laboratory‐scale into large‐scale reactions is crucial in making technology‐based magnetoelectrochemistry a core solution in climate change discussions. To this end, the future deployment of device‐based magnetoelectrochemistry in any chemical transformation process also requires a deep understanding of other induced effects such as chemical and thermal stability under a magnetic field with extended exposure time and assessments of energy consumption and viability.

## Conflict of interest

The authors declare no conflict of interest.

## Biographical Information

*Songzhu Luo is currently a PhD candidate in the School of Materials Science and Engineering, Nanyang Technological University (Singapore). He obtained his B.Eng. degree from Central South University (China) in 2019. His current research interests focus on electrocatalysis for energy conversion*.

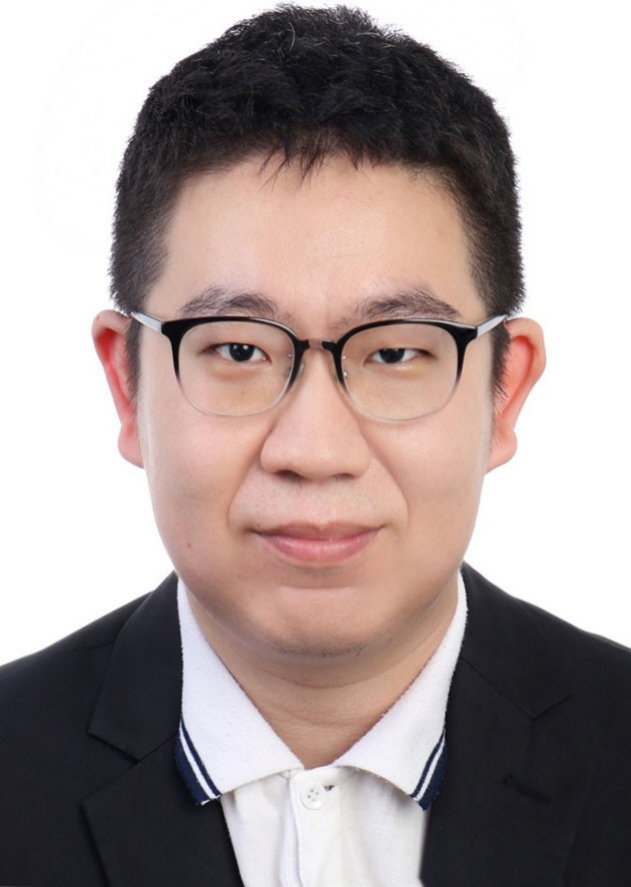

## Biographical Information

*Kamal Elouarzaki received his PhD degree in 2011 from Moltech‐Anjou (Angers University, France), and he was a CNRS postdoctoral fellow at the University of Joseph Fourier, Grenoble (France), from 2011 to 2014. He spent four years at the Cambridge Centre for Advanced Research and Education in Singapore. In 2021, he took up a position as a senior scientist at Nanyang Environment & Water Research Institute in Singapore*.

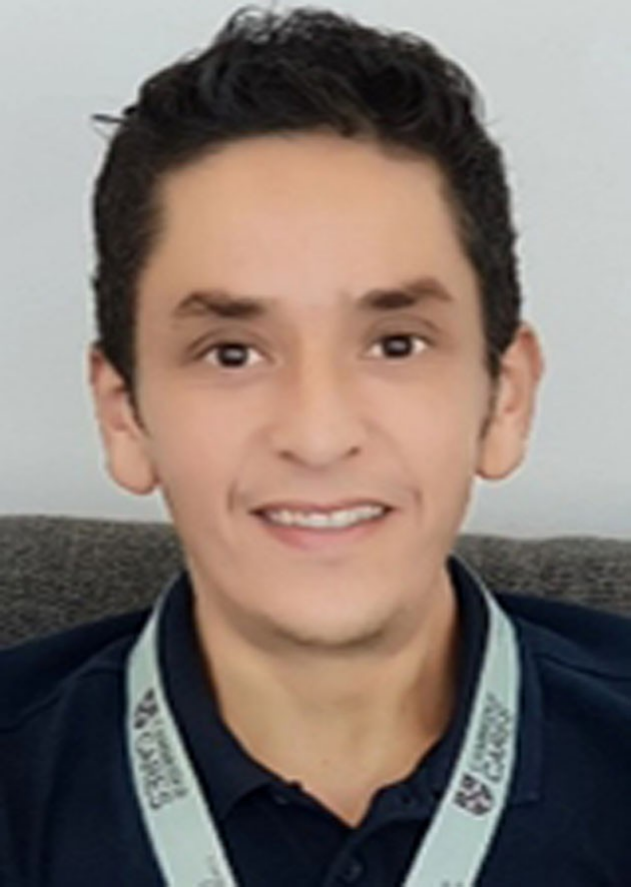

## Biographical Information

*Zhichuan J. Xu is a professor at the School of Materials Science and Engineering, Nanyang Technological University (Singapore). He is a member of the International Society of Electrochemistry (ISE), The Electrochemistry Society (ECS), and a Fellow of the Royal Society of Chemistry (FRSC). He serves as the president of ECS Singapore Section. His major research interest is electrocatalysis*.